# *Bacteroides dorei* dominates gut microbiome prior to autoimmunity in Finnish children at high risk for type 1 diabetes

**DOI:** 10.3389/fmicb.2014.00678

**Published:** 2014-12-10

**Authors:** Austin G. Davis-Richardson, Alexandria N. Ardissone, Raquel Dias, Ville Simell, Michael T. Leonard, Kaisa M. Kemppainen, Jennifer C. Drew, Desmond Schatz, Mark A. Atkinson, Bryan Kolaczkowski, Jorma Ilonen, Mikael Knip, Jorma Toppari, Noora Nurminen, Heikki Hyöty, Riitta Veijola, Tuula Simell, Juha Mykkänen, Olli Simell, Eric W. Triplett

**Affiliations:** ^1^Department of Microbiology and Cell Science, Institute of Food and Agricultural Sciences, University of FloridaGainesville, FL, USA; ^2^Department of Pediatrics, Turku University Hospital and University of TurkuTurku, Finland; ^3^Department of Pediatrics, University of FloridaGainesville, FL, USA; ^4^Department of Pathology, Immunology and Laboratory Medicine, University of FloridaGainesville, FL, USA; ^5^Department of Clinical Microbiology, University of Eastern FinlandKuopio, Finland; ^6^Immunogenetics Laboratory, University of TurkuTurku, Finland; ^7^Department of Pediatrics, Children's Hospital, University of Helsinki and Helsinki University Central HospitalHelsinki, Finland; ^8^Diabetes and Obesity Research Program, University of HelsinkiHelsinki, Finland; ^9^Department of Pediatrics, Tampere University HospitalTampere, Finland; ^10^School of Medicine, University of TampereTampere, Finland; ^11^Department of Pediatrics, University of Oulu, and Oulu University HospitalOulu, Finland

**Keywords:** *Bacteroides dorei*, *Bacteroides vulgatus*, type-1 diabetes, autoimmunity, microbiome

## Abstract

The incidence of the autoimmune disease, type 1 diabetes (T1D), has increased dramatically over the last half century in many developed countries and is particularly high in Finland and other Nordic countries. Along with genetic predisposition, environmental factors are thought to play a critical role in this increase. As with other autoimmune diseases, the gut microbiome is thought to play a potential role in controlling progression to T1D in children with high genetic risk, but we know little about how the gut microbiome develops in children with high genetic risk for T1D. In this study, the early development of the gut microbiomes of 76 children at high genetic risk for T1D was determined using high-throughput 16S rRNA gene sequencing. Stool samples from children born in the same hospital in Turku, Finland were collected at monthly intervals beginning at 4–6 months after birth until 2.2 years of age. Of those 76 children, 29 seroconverted to T1D-related autoimmunity (cases) including 22 who later developed T1D, the remaining 47 subjects remained healthy (controls). While several significant compositional differences in low abundant species prior to seroconversion were found, one highly abundant group composed of two closely related species, *Bacteroides dorei* and *Bacteroides vulgatus*, was significantly higher in cases compared to controls prior to seroconversion. Metagenomic sequencing of samples high in the abundance of the *B. dorei/vulgatus* group before seroconversion, as well as longer 16S rRNA sequencing identified this group as *Bacteroides dorei*. The abundance of *B. dorei* peaked at 7.6 months in cases, over 8 months prior to the appearance of the first islet autoantibody, suggesting that early changes in the microbiome may be useful for predicting T1D autoimmunity in genetically susceptible infants. The cause of increased *B. dorei* abundance in cases is not known but its timing appears to coincide with the introduction of solid food.

## Introduction

Type 1 diabetes (T1D) is an autoimmune disorder typically developing within the first 5–6 years of life, beginning with inflammation of the pancreas, progression to autoimmunity and ultimately destruction of insulin-producing beta cells in the pancreas, resulting in dependence on daily exogenous insulin to control blood glucose levels. T1D is a complex disorder whose etiology is poorly understood, but it is widely believed that a combination of genetic and environmental factors contribute to increased T1D risk (Knip et al., 2005). Disease incidence rate varies dramatically by country with Finland having the highest known rate (Karvonen et al., 1993). While genetics explains 50% of T1D risk (Atkinson et al., 2014), the acceleration in this disease's incidence over the last 50 years in developed countries strongly suggests that T1D propensity is increasingly due to environmental factors (Karvonen et al., 1993).

Evidence from both rodent models and human studies suggests that the composition of the gut microbiome has a significant influence on the immune system development and may influence T1D risk. Feeding of antibiotics (Brugman et al., 2006; Schwartz et al., 2007) or a probiotic-like bacterial strain (Valladares et al., 2010) reduced autoimmune diabetes in disease-prone rodent models. Non-obese diabetic (NOD) mice raised in germ-free environments have an increased risk for diabetes (King and Sarvetnick, 2011) while exposure to bacterial antigens and infection decrease T1D risk (Bach, 2002). NOD mice that lack an adaptor for multiple innate immune receptors have an altered microbiome compared to control strains (Wen et al., 2008). In rats with diabetes, a culture-independent study showed increased proportions of *Bacteroides* and decreased *Lactobacillus*, compared to control rats (Roesch et al., 2009b). In humans, gut microbiome analysis of small cohorts of T1D autoimmune and control children showed significant bacterial taxa and functional differences (Brown et al., 2011; Giongo et al., 2011; de Goffau et al., 2013). More recently, a large case-control study from the BABYDIET study cohort (Schmid et al., 2004; Hummel et al., 2011) showed that microbial interaction networks were impaired in case samples, although no significant differences between the abundances of bacterial taxa were identified in cases versus controls (Endesfelder et al., 2014).

The evidence of a microbial role in the development of Type-1 diabetes lead to the formation of several studies on human cohorts including TEDDY (TEDDY Study Group, 2008), BABYDIET, and this study. These studies share several features including being retrospective, longitudinal across age, and controlling for genotype by selecting only subjects at moderate to high genetic risk. This study is the largest published study to date in terms of numbers of samples as well as subjects and is the only study where all subjects were from the same city and born in the same hospital thus controlling for many environmental factors which may confound studies of T1D and the microbiome. Results reported here from a large Finnish cohort support an association of a single species of bacteria, *Bacteroides dorei*, in the development of T1D autoimmunity in children at high risk for the disease. While this association does not establish a causative role in T1D development, observed changes in the gut microbiome occurred before development of the disease and could potentially be used as a biomarker for early screening.

## Materials and methods

### IRB approval

Prior to commencing with this work, the University of Florida's Institutional Review Board assessed and approved this work as an exempt project with number 278-2010.

### Collection of stool samples

The Finnish Type 1 Diabetes Prediction and Prevention Study (DIPP) began genetic screening of newborn infants from the general population in 1994 (Nejentsev et al., 1999). Newborns were screened for high-risk HLA-DR and HLA-DQ genotypes using a previously described method (Nejentsev et al., 1999). Monthly stool samples were collected by the subjects' parents at home and mailed to the DIPP Virus Laboratory for virology in Tampere, Finland, where they were stored at −20 to −80°C. In this study, 76 subjects were retroactively selected to create a cohort of age-matched genotype-controlled subjects for the investigation of the microbiome as an environmental factor influencing risk of autoimmunity.

Subjects were born between 1996 and 2007 at the Turku University Hospital, Turku located in southwestern Finland (Table S1). A total of 947 stool samples were collected between 1996 and 2010 at monthly intervals when subjects were between 4–6 months old until to 2.2 years old (Table S2). Mode of delivery, duration of exclusive and total breast feeding, and antibiotics use were recorded (Tables S1, S4). As all samples were collected from within 80 km of the Turku University Hospital, the time from stool collection to frozen storage was likely not long. Previous work with Turku stool samples has shown minimal change to the microbial community if frozen within 24 h (Roesch et al., 2009a).

Detection of anti-islet autoimmunity was performed as described in Parikka et al. (2012). According to the DIPP data collected by 30 November 2011, the following groups of children with available stool samples were included in the analyses: cases (*n* = 29) who developed at least two persistent T1D-associated autoantibodies of whom 22 (75%) progressed to T1D later during the follow-up and clinically unaffected healthy control children (*n* = 47) without any auto-antibodies (Table S1). These 47 subjects in the study did not express any islet autoantibodies during the course of the study and are from here referred to as controls.

Several known confounders of microbiome studies including breast feeding (total duration/any), duration exclusive breast feeding, mode of delivery, and antibiotic use (including amoxicillin and azithromycin, and 7 others) were compared between case and control groups via the chi-square test (Table S5, see Supplementary Methods) to ensure that these variables were balanced between cases and controls.

### DNA extraction, 16S rRNA and metagenomic sequencing

DNA was extracted from approximately 200-mg of stool sample using Qiagen AllPrep DNA/RNA/Protein Mini Kit (QIAGEN). Precautions were taken to avoid contamination, and blank amplification controls were processed adjacent to samples. DNA was quantified using a Nanodrop spectrophotometer (Thermo Scientific, Wilmington, DE). The primers used for 16S rRNA amplification and subsequent Illumina HiSeq sequencing were the V3 and V4 universal (Caporaso et al., 2011). For MiSeq sequencing, the V3 primer was replaced by the 314F primer (Muyzer et al., 1996). Amplification 16S rRNA gene was performed as described previously (Fagen et al., 2012). PCR reactions were prepared with 25-μ l of GoTaq colorless Master Mix (Promega, Madison, WI), 10-μ M of primers, 20-ng of sample DNA adjusted to 50-μ l total volume with sterile, nuclease-free water. Samples were subjected to an initial denaturation temperature of 95°C for 3 min, followed by 25 cycles of 95°C for 45 s, 53°C for 30 s, and 73°C for 90 s, and a final elongation at 73°C for 10 min. Amplified DNA was quantified fluorometrically with Qubit dsDNA High Sensitivity (Invitrogen, Life technologies Inc., Carlsbad, CA), and fragment size was verified on a gel. Neither DNA nor amplification was detected in blank control samples. Samples were randomly distributed across barcodes, lanes and sequencing runs to avoid any systematic bias. Equal masses of DNA from barcoded amplicons were merged and sequenced on the Illumina HiSeq 2000, and MiSeq platforms (Table S3).

### Stool 16S rRNA sequencing using 2 × 100 bp illumina hiseq

A total of 113 gigabases of nucleotide data were generated using three plates and seven lanes of Illumina HiSeq-2000 sequencing. Sequences were de-multiplexed based on the barcode sequence and reads with unmatched barcodes were discarded. Reads were trimmed for quality to remove bases with a high probability of error using the program Sickle (Joshi, 2013), and reads containing ambiguous nucleotides or that were shorter than 70 nucleotides after trimming were discarded. The 3′-most sequence in each pair was reverse-complemented so that all sequences were in the 5′–3′ orientation. Removal of low quality bases as well as reads that contained ambiguous nucleotides resulted in an average of 375,313 (*SE* = 5107) reads per sample with an average length of 98 ± 3 nucleotides. This length is adequate to assign reads to genus level (Liu et al., 2007). By discarding samples with fewer than 10,000 reads (*n* = 3) after trimming, 947 samples remained with a average of 357,581 (*SE* = 6400) reads per sample.

### Microbiome 16S rRNA gene analysis

To assign and quantify the Operational Taxonomic Units (OTUs) present in each stool sample, sequences were aligned to the GreenGenes 97% representatives set version 13.5 (DeSantis et al., 2006) using the USEARCH program version 6.022 (Edgar, 2010), a component of QIIME (Caporaso et al., 2010), with 97% minimum identity and 95% minimum query coverage. In order to account for differences in sampling depth, read counts were subsampled using rarefaction with a sample size of 10,000 reads then divided by the total to calculate the percent relative abundance.

**Figure 1:**
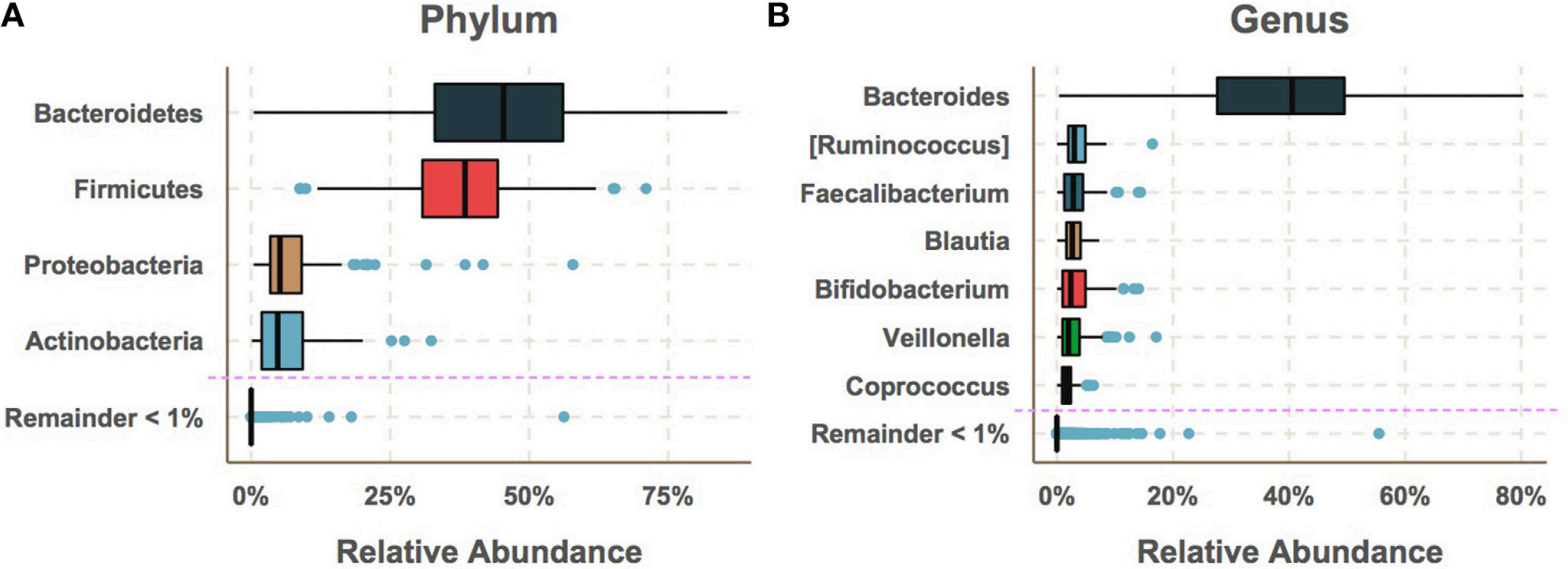
**The gut microbiome had low diversity and was dominated by a few taxa with median relative abundances greater than 1%**. **(A)** The most abundant phyla were Bacteroidetes and Firmicutes followed by Proteobacteria and Actinobacteria. **(B)** The most abundant genus was by far *Bacteroides* followed by *Ruminococcus*, *Faecalibacterium*, *Blautia*, *Bifidobacterium Veillonella* and *Coprococcus*. While a large number of species were detected, the majority of these had very low relative abundance.

A total of 335 technical replicates were used to measure variation due to DNA extraction, amplification and differences in sequencing run and lane and sequencing technology (Table S3). Variance of relative abundance within technical replicates was found to sharply increase as relative abundance decreased (see Supplementary Methods). This trend was used to define a cutoff for minimum relative abundance below which accurate estimation of relative abundance is not possible. This cutoff was set at 1% relative abundance and taxa below the 99% most-abundant taxa were excluded from the analysis due to their having high variance within technical replicates for DNA extraction, amplification and sequencing (Tables S6–S8). These filtered taxa made up a small portion of the total gut microbiome (Figures 1A,B). However, the total number of taxa including those below the cutoff was taken into account when performing the false discovery rate (FDR) correction.

### Statistical comparison of case and control microbiomes

The relative abundances of taxa at ranks Phylum, Genus, and Species were compared between all samples from all cases and controls using the non-parametric Mann–Whitney *U*-test (Tables S6–S8). To adjust for differences in per-subject sampling frequency, *p*-values were averaged over 100 bootstrap iterations with even per-subject sampling frequency and bootstrapped *p*-values were adjusted for FDR using the B-H method (Benjamini and Hochberg, 1995) using the total number of taxa including those that fell below 1% average relative abundance. Significance was considered if the adjusted, bootstrapped *p*-value fell below alpha = 0.001.

**Figure 2:**
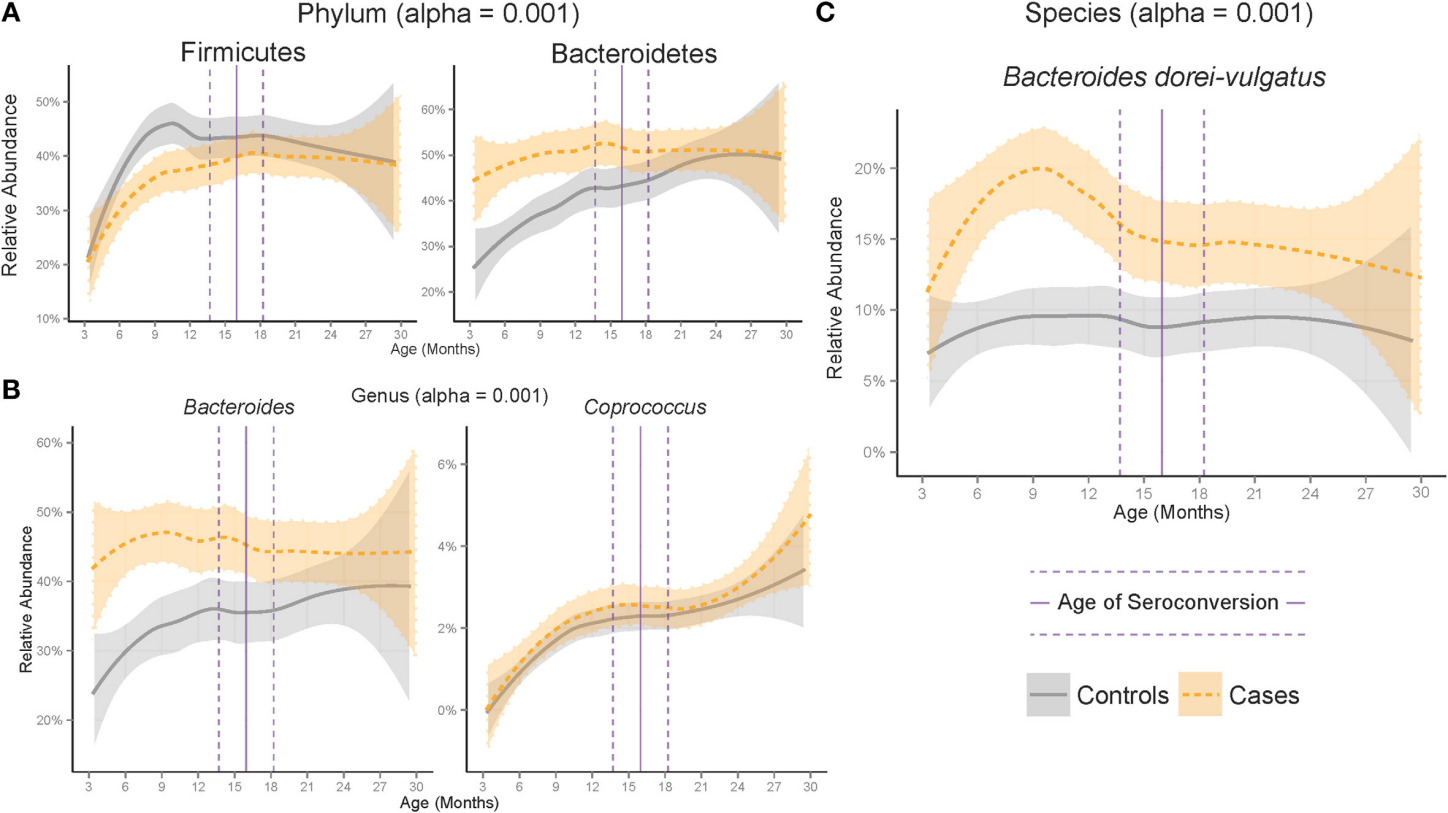
**Development of the microbiome differed between cases and controls at the (A) Phylum, (B) Genus and (C) Species ranks across age**. Plotted are significant taxa with median relative abundance greater than 1%. Shaded region represents standard error of a LOESS smoothing regression. Vertical lines represent the median (solid) and standard error (dashed) age of appearance of first the autoantibody in cases.

Logistic regression was used to model the interaction of age and relative abundance of each taxon with seroconversion as the response variable and model terms were tested for significant association with seroconversion. In this case, the model terms were the interaction between age and relative abundance. Significance of model terms was tested for using ANOVA. Model terms that fit significantly to seroconversion can be said to significantly vary between cases and controls. FDR-correction was applied to *p*-values using the same method as the Mann-Whitney U test. The relative abundances of significant taxa were visualized across age using LOESS regression (Figure 2).

### Metagenomic sequencing of samples enriched for *Bacteroides dorei*

DNA samples with a *Bacteroides* abundance above 47%, as measured by 16S rRNA analysis, were sequenced by the Pacific Bioscience RS-II platform with data assembled and analyzed as described previously (Leonard et al., 2014). Two of these four metagenomes used in this work were closed previously (Leonard et al., 2014, NCBI accession numbers CP007619 and CP008741). A third closed *B. dorei* genome is described here (NCBI accession number CP009057) which was obtained in the same manner from a healthy child, sample number 728. A fourth metagenome from a case subject, sample number 233 resulted in a partially assembled *B. dorei* genome. All four metagenomes were mined for 16S and 23S rRNA using RNAmmer (Lagesen et al., 2007), as well as for a beta-glucosidase gene that has been reported to be present in *B. dorei* but lacking in *B. vulgatus* (Pedersen et al., 2013).

**Figure 3:**
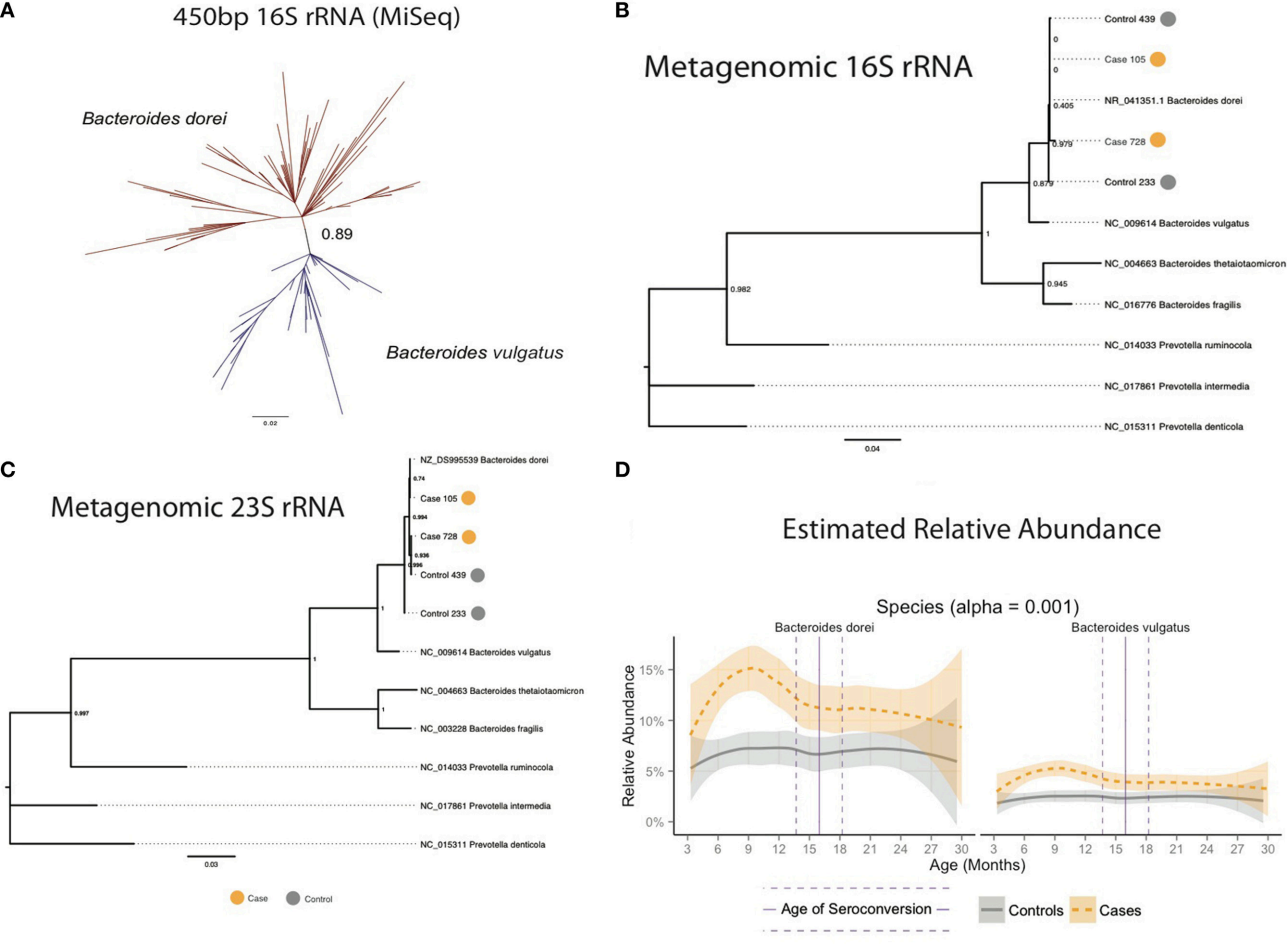
**(A)** Longer 16S rRNA MiSeq reads separated *Bacteroides dorei* from *B. vulgatus*. **(B)** Comparison of full-length 16S and **(C)** 23S rRNA genes from closed and partially closed *B. dorei* genomes from stool shotgun metagenomes identified the *B. dorei/vulgatus* group as *B. dorei*. and **(D)** *B. dorei/vulgatus* group appears to be dominated by *B. dorei*.

The 16S and 23S rRNA sequences mined from the metagenomic DNA were combined with sequences from *Bacteroides* species using *Prevotella* as an outgroup, aligned using MUSCLE and used to generate phylogenetic trees with PhyML using a GTR model (Figure 3A). These trees had nodes with higher (greater than 0.80) bootstrap support value and were capable of confidently separating *B. dorei* from *B. vulgatus*.

### Identification of *Bacteroides dorei* as the main component of the *Bacteroides dorei/vulgatus* group

The length of the 2 × 100 bp Illumina HiSeq reads was insufficient to accurately distinguish *B. dorei* from *B. vulgatus* (Figure S1). Additional sequencing with overlapping 2 × 350 bp reads on the Illumina MiSeq platform to obtain assembled 450 bp reads was conducted on a set of 93 samples with >10% relative abundance of *Bacteroides* to improve taxonomic resolution of the *B. dorei*/*B. vulgatus* group. Reads were generated using a different forward primer (Muyzer et al., 1996), that targeted the 314–357 F region of the 16S rRNA gene. Overlapping MiSeq reads were assembled into single contigs using Pandaseq (Masella et al., 2012) and filtered to include only reads longer than 450 bp. Assembled MiSeq reads were trimmed to exactly 450 bp. A subset of 10,000 reads from each sample were clustered using *de novo* clustering at 99.6% identity by UCLUST (Edgar, 2010) to identify representative taxa. Representative sequences were combined with truncated 16S rRNA sequences from known *Bacteroides* species from GreenGenes and 16S rRNA sequences from the four PacBio metagenomes, aligned with MUSCLE (Edgar, 2004), and used to create a phylogenetic tree using PhyML with the GTR model (Guindon et al., 2005). A high bootstrap value (0.89) was measured in the branch separating *B. dorei* and *B. vulgatus* (Figure 3C). Representative sequences were then re-labeled as *B. dorei* and *B. vulgatus* depending on their placement in the phylogenetic tree and used as references to align the MiSeq reads to estimate relative abundance of the two groups and compared to the combined *B. dorei* and *B. vuglatus* relative abundance obtained from shorter reads.

Poisson linear regression was used to estimate the relative abundance of *B. dorei* and *B. vulgatus* based on the correlation between HiSeq relative abundance of the *B. dorei/vulgatus* group and MiSeq relative abundance of *B. dorei* and *B. vulgatus* separately (see Supplementary Methods). Samples were up-weighted based on relative abundance due to their measured increase in variance resulting in linear regressions with good fit (*R*^2^ = 0.94). Fitted linear regressions were then used to estimate *B. dorei* and *B. vulgatus* relative abundance for the remaining samples based on HiSeq data (Figure 2D).

### Determining timing of *Bacteroides dorei* spike

To determine the specific timing of the *B. dorei* spike in cases, samples were binned into windows across age with average width of 98 days (*SE* = 0.33), two samples per individual and 98 samples per window (*SE* = 2.98). Windows with less than 20 cases and 20 controls were discarded. Downsampling was used to account for differences in subject sampling frequency by taking the median relative abundance for each taxon from multiple samples from the same individual. Bootstrapping was used to account for differences in sampling frequency between cases and controls averaging Mann-Whitney U *p*-values over 100 bootstraps within each window (Figure 3, Table S5).

### Estimation of absolute *Bacteroides dorei* abundance

Sequencing of 16S rRNA amplicons can only measure relative (proportion of the total) abundance. Therefore, the spike in relative abundance of *B. dorei* could be either a true spike in number of *B. dorei* cells or a decrease in the abundance of all other taxa. This leaves two possibilities, either *B. dorei* is associated with disease or the reduction in all of the other bacterial inhabitants are associated with disease but *B. dorei* remains. The total abundance of *B. dorei* was measured in order to distinguish the two possibilities. SYBR Green Quantitative PCR (qPCR) was used to estimate absolute abundance of the *Bacteroides* group using *Bacteroides*-specific primers that were shown to have high sensitivity and specificity (Liu et al., 2003). Twenty-eight samples taken from subjects between the age of 200 and 350 days were chosen based on (1) equal number of cases and controls; (2) good representation of the range of relative abundances; and (3) high DNA concentration (20 ng/uL). Amplification of a standard curve of PCR product generated from *Bacteroides fragilis* ATCC 25285 cDNA using the same primers in triplicate was used to calculate copy number in the stool DNA. Six samples were thrown out due to having high intra-triplicate variance. Relative abundance and absolute abundance were highly correlated using a Poisson linear regression (*p* = 2.18 × 10^−35^, see Supplementary Methods).

### Predicting seroconversion based on *Bacteroides dorei* relative abundance

The accuracy of a logistic regression model to measure the accuracy of predicting seroconversion based on the relative abundance of *B. dorei* was estimated using leave-one-subject-out cross-validation (Table S5). Because of the possibility of the logistic regression classifying a subject as case or control could depend on recognition of that subject's microbiome rather than true differences between cases and controls, relative abundances were downsampled per subject and no sample from the same subject was used for training and testing. Cross-validation accuracy was measured using the Area Under the receiver operating Characteristic (AUC) with significance being measured using an analysis of variance with a chi-squared test (Table S5).

### Availability of source code and data

Source code used for the statistical analyses is available at https://github.com/triplett/dipp-microbiome-study. The 16S rRNA sequencing reads for this work are archived in NCBI BioProject PRJNA232731. The *B. dorei* genome sequenced in this study (from sample 728) is available under the NCBI accession CP009057. The Illumina HiSeq and MiSeq 16S sequences are available from NCBI's Short Read Archive under Study SRP043574. Table S10 contains a table of unrarefied 16S rRNA read counts at Phylum, Genus and Species ranks.

Analyses were performed using functions in the R programming language (R Core Team, 2012) as well as the Phyloseq (McMurdie and Holmes, 2012) and ggplot2 (Wickham, 2009).

## Results

To examine the development of the gut microbiome in children at genetic risk for type 1 diabetes (T1D), DNA from 947 stool samples from 76 number children having the moderate (odds-ratio <5) to high risk (odds-ratio 5–15) for T1D according to their HLA-DQB1 genotype, which is known to increase T1D risk, was sequenced. The HLA-DQ genotype is the most common high-risk genotype for T1D and confers a greater risk for future T1D than other high-risk alleles (Barrett et al., 2009). Twenty-nine subjects developed at least three persistent T1D-relative autoantibodies (mean age at diagnosis = 16.8 months, *SE* = 2.03), which collectively confers >95% risk of clinical T1D diagnosis. Those 29 subjects are considered “cases” for the purposes of this study. The remaining 47 subjects with no detectable T1D-related autoantibodies in serum were retroactively selected to serve as “controls” (Table S1). Genetic risk for T1D was balanced between case and control groups leaving the remaining risk up to environmental factors. To better control for environmental variables other than the gut microbiome including factors which are known to effect the microbiome such as mode of delivery (Dominguez-Bello et al., 2010), breast feeding (Cabrera-Rubio et al., 2012), and antibiotics (Dethlefsen et al., 2008; Jakobsson et al., 2010; Panda et al., 2014), subjects recruited for the retrospective, longitudinal DIPP study were examined for differences in these factors. None of these factors were significantly different between cases and controls (*p* > 0.05, see Supplementary Methods). No information on introduction of solid food was collected. However, months of exclusive breast feeding did not differ between cases and controls. All subjects were born in the Turku University Hospital in Turku, Finland allowing us to specifically examine differences in the gut microbiome compositions of individuals progressing to T1D-related autoimmunity vs. those not developing T1D-autoimmunity, while controlling for other environmental factors.

### Gut microbiome differs between cases and controls before seroconversion

To determine the extent to which gut bacterial communities may differ between genetically at-risk subjects progressing toward T1D and those avoiding T1D progression, bacterial community 16S rRNA genes were sequenced from 947 stool samples collected every 36 days (*SE* = 11) on average (Table S2) and were assigned sequences to “operational taxonomic units” (OTUs) in order to characterize the taxonomic composition of each sample's community. At the phylum level, the gut microbiomes were dominated by Bacteroidetes and Firmicutes followed by Actinobacteria and Proteobacteria (Figure 1A). At the genus level, these samples were high in relative abundance of *Bacteroides*(30–50%) followed by moderate levels(<10%) of *Ruminococcus*, *Faecalibacterium*, *Blautia*, *Bifidobacterium*, *Veillonella*, and *Coprococcus* (Figure 1B). Taxa below 1% median relative abundance were filtered from the analysis as the abundance measurement of these taxa was unreliable in technical replicates (see Supplementary Methods). The remaining dominant taxa made up the vast the majority of the net microbial community consistent with previous studies on the human gut microbiomes of healthy and at-risk humans (Brown et al., 2011; Giongo et al., 2011; Kraal et al., 2014).

Gut microbiomes were compared between cases and controls by testing for differences in relative abundance across all samples and across age (see methods). All statistical tests were adjusted for false discovery rate, a common problem in studies with a high-number of variables. Taxa below 1% were included in the FDR correction. Microbiome differences were only considered significant if their adjusted *p*-values fell below an alpha level of 0.001. This approach is likely to make our approach conservative, while focusing on the high-abundance taxa more likely to have large effects on biological processes in the human gut.

Across all samples, the Gram-negative phylum Bacteroidetes was on average 6.7% higher in cases than controls (adjusted *p* = 2.17e-6) and the Gram-positive phyla Proteobacteria and Tenericutes were significantly higher in controls (adjusted *p* = 1.59e-7 and 7.20e-8 respectively). Comparison of the gut microbiome across age at showed only Bacteroidetes and Firmicutes to be significantly different between cases and controls (Figure 1A, adjusted *p*-values = 1.35e-10, 2.2e-5, respectively). Further examination of the gut microbiome communities of cases vs. controls revealed that a single dominant genus *Bacteroides* was significantly higher in cases vs. controls using all samples at all time points with a median relative abundance of 44.4% in cases (*SE* = 5.5 × 10^−3^) and 30.7% (*SE* = 10.2 × 10^−3^) in controls (adjusted *p* = 1.6e0-7). *Bacteroides* and *Coprococcus* were found to vary significantly across age (Figure 2B, adjusted *p*-values = 3.6e-11, 8.5e-05, respectively) although *Coprococcus* was only significantly higher in controls at a higher alpha considered in this study (adjusted *p* = 0.034).

The *Bacteroides* genus was found to be dominated by two closely related species, *Bacteroides dorei* and *Bacteroides vulgatus*, which differed significantly between cases and controls across all samples, across age (*p* = 1.78e-17, Table S3) and within several windows prior to seroconversion (Figure 1C, *p* < 0.01, Table S5). These two species were not distinguishable by their V4 16S rRNA region preventing further identification (see methods). Longer sequencing of the combined V3 and V4 regions in 93 samples differentiated the two species identifying *B. dorei* as the major constituent and both *B. dorei and B. vulgatus* (Figure 3C).

To provide further evidence of the identity of the *B. dorei/vulgatus* group as *B. dorei*, we performed shotgun metagenomic sequencing on four stool samples and succeeded in the closure of three genomes closely resembling *B. dorei, two* of which we reported previously (Leonard et al., 2014). These genomes had higher average nucleotide identity to *B. dorei* than *B. vulgatus*, as well as a beta-galactosidase gene which *B. vulgatus* lacks (Bakir et al., 2006). Assembly of the fourth genome did not result in genome closure. However, full-length 16S and 23S rRNA genes mined from all four samples clustered at high confidence with *B. dorei* on a phylogenetic reconstruction (Figures 3A,B). Relative abundance of *B. dorei* in these metagenomes closely matched that measured by 16S rRNA sequences (*R*^2^ = 0.97, Table S9).

**Figure 4:**
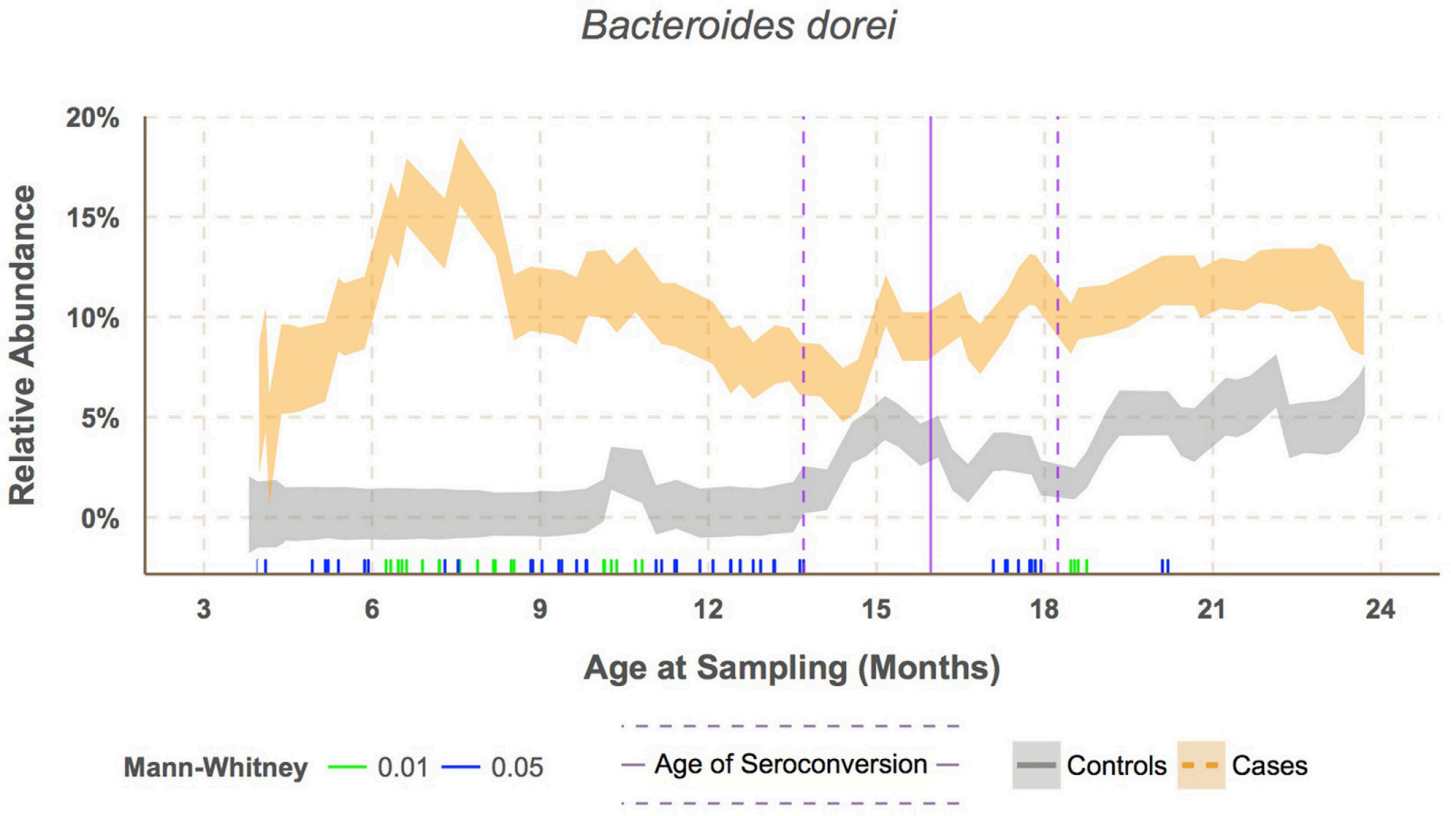
**Sliding window analysis showed *Bacteroides dorei* being significantly higher in cases prior to seroconversion at around 7.6 months of age**. Vertical bars along the bottom of the x-axis represent median subject age in windows with significantly different relative abundance between cases and controls at alpha levels 0.05 (green) and 0.01 (blue) as determined by the Mann–Whitney test. Relative abundance was downsampled by subject within each window to prevent one subject's microbiome from dominating the results.

*B. dorei* made up roughly 75% of the *B. dorei/vulgatus* group (Figure 3D, see methods) and was significantly higher in cases across age using the logistic regression approach (*p* < 0.001). *B. dorei* was 5 times more abundant in cases than controls across all samples and reached up to 100 times higher in cases (17.3% relative abundance, *SE* = 0.02) versus controls (0.18%, *SE* = 0.01) within a 78-day window at 7.5 month of age, or 9.2 months prior to the average age of first T1D autoantibody (*p* = 0.005, Figure 4, Table S5).

High-throughput sequencing is limited by its ability to only measure relative as opposed to absolute abundance. As a result, an apparent increase in the relative abundance of a taxon could be the result of either an actual increase in absolute abundance of that taxon or a decrease in all remaining taxa. To overcome this limitation, quantitative PCR was done on 22 stool DNA samples using universal *Bacteroides*-specific primers. The relative abundance of *Bacteroides* was highly correlated with absolute abundance (see Supplementary Methods). Therefore, this increase in *B. dorei* is likely due to an increase in actual number of cells, not a decrease in all other taxa.

**Figure 5:**
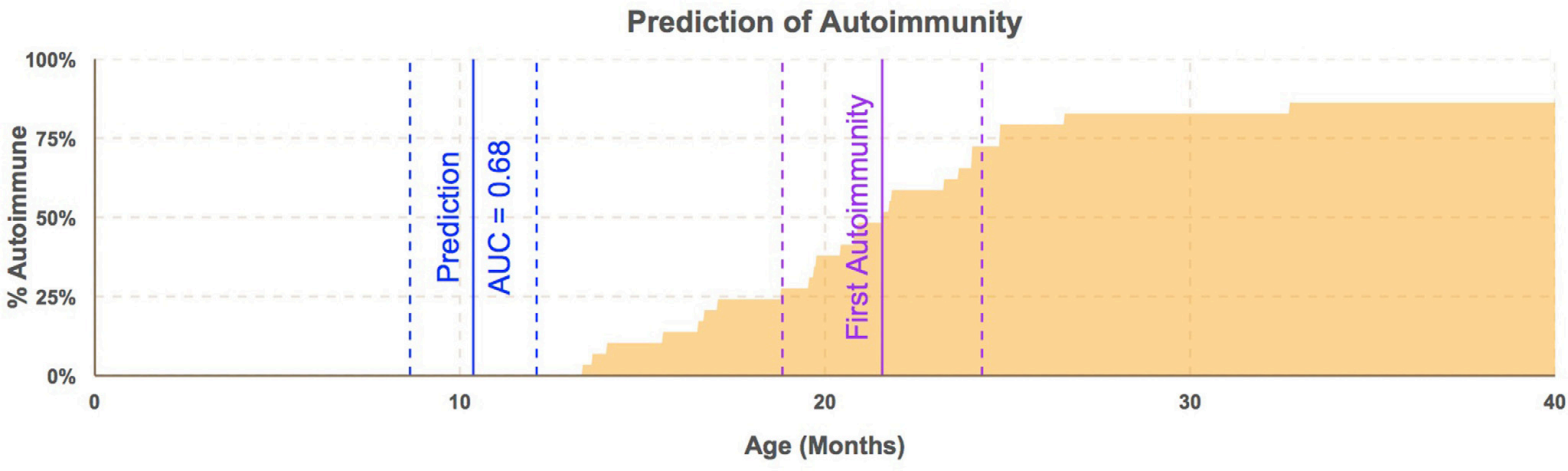
***Bacteroides dorei*/*vulgatus* predicts autoimmunity before appearance of any single autoantibody**. Timing of prediction of autoimmunity. Blue line represents median age (11 months) of subjects in windows of samples taken from subjects aged 6.1–12.1 months with standard error lines (dashed). The purple line represents median age of the appearance of the first autoantibody with standard error lines (dashed). The proportion of cases who have seroconverted is plotted across time (orange area). Estimation of the efficacy of *B. dorei* to serve as a predictor of future autoimmunity was significantly better than chance within this window (AUC = 0.68, *SE* = 0.007) before the appearance of any single autoantibody.

To test whether the *B. dorei* can be used as a biomarker to predict future autoimmunity and type-1 diabetes, we tested efficacy of classification of case/control status based on *B. dorei* abundance using cross-validation, which estimates a predictor's accuracy on real-world data. Cross-validation was performed using the sliding window across age and accuracy was found to peak in samples taken between 6.1 and 12.1 months of age, between 12 and 15 months before the first detection of T1D autoimmunity (Figure 5, Table S5). Accuracy measured by the Area Under the Curve (AUC) to be 0.69 (*SE* = 0.007) which was significantly higher than chance (*p* = 0.019, Figures 4, 5, Table S5) using data downsampled by subject in order to avoid overfitting to subjects' microbiomes. However, a true, blind leave-out dataset is required in order to measure the accuracy of microbiome-based prediction of autoimmunity on real-world data (Babyak, 2004).

## Discussion

In this cohort, high levels of *Bacteroides dorei* in the gut are associated with future autoimmunity for T1D. *Bacteroides dorei* is a recently discovered species isolated from the human gut (Bakir et al., 2006) and may be mistaken with *B. vulgatus* due to their taxonomic similarity (Bakir et al., 2006). However, whole genome analysis shows that the two species are sufficiently different to warrant separate species designations. Nevertheless, the two species are very similar and have not yet been distinguished from each other physiologically apart from beta-galactosidase activity in *B. dorei* (Bakir et al., 2006).

Higher levels of *Bacteroides* in cases were observed previously in a rat model for T1D as well as in human cohorts (Roesch et al., 2009b; Brown et al., 2011; Giongo et al., 2011). However, this work advances our knowledge in three ways. First, it is the largest human T1D cohort examined to date and is a cohort derived from a single city with all children born in the same hospital. Second, a frequent sampling of the subjects occurred from about 6 months after birth until 3 years of age, which provides the ability to observe the development of the microbiome across age and identify potential associations. And third, the analyses performed here suggest a role for a single, dominant bacterial species associated with T1D autoimmunity.

Increased abundance of *Bacteroides* species including *B. dorei* and *B. vulgatus* have been implicated in inflammation in several gut diseases such as ulcerative colitis (UC), irritable bowel disease (IBD), and celiac disease (CD) (Breeling et al., 1988; Rath et al., 1999; Fujita et al., 2002; Sanchez et al., 2010, 2011; Sato et al., 2010; Schippa et al., 2010; Bloom et al., 2011). A recent report showed that *B. vulgatus* was found in duodenal biopsy specimens of 70% of subjects (14 of 20) with untreated CD versus 0% of subjects (0 of 12) treated with a gluten-free diet for at least two years (Schippa et al., 2010).

While it has not been shown whether *B. vulgatus* or *B. dorei* are causative rather than correlated with disease, *B. vulgatus* induces colitis in rats, mice, and guinea pigs (Salyers et al., 1977; Fujita et al., 2002; Sanchez et al., 2011). Strains of *B. vulgatus* associated with human UC have a rougher surface and higher cell adherence rates than *B. vulgatus* strains derived from non-UC patients (Schippa et al., 2010). *B. vulgatus* is not thought to directly cause IBD but may prevent its remission (Fujita et al., 2002) and is also known as an opportunistic pathogen causing intra-abdominal infections (Rath et al., 1999). Infants with high-risk HLA genotypes for T1D and CD had higher prevalence of *B. vulgatus* than infants at low genetic risk (Sanchez et al., 2011). *B. vulgatus* was more common in CD patients, and *B. dorei* was more common in patients with active CD (Sanchez et al., 2010).

The cause of increased *B. dorei* in these subjects is not known. *Bacteroides* are observed in infants after the first introduction of solid food (Nwaru et al., 2010). *Bacteroides* colonization of the human gut at this early stage of life is important given their role in the digestion of complex carbohydrates (Salyers et al., 1977). At the time of the infant period for the current study participants, the national health care system in Finland recommended that parents should introduce solid food between the age of 4 and 6 months (Simell and Aula, 1997; Hasunen et al., 2004). Thus, the appearance of a large proportion of *Bacteroides* in these children at 6 months of age appears to be associated with the introduction of solid food.

*Bacteroides* possess high numbers of antibiotics resistance genes. It is possible that the increase in antibiotic usage, especially in developed countries, has led to an overall increase in *Bacteroides* across entire populations and in their environment. This might explain the recent increase in T1D prevalence as well as country-by-country differences. This study lacks sufficient data to estimate the effect of antibiotics on *Bacteroides* abundance in the gut and risk for autoimmunity and T1D. Larger cohorts with an antibiotic control group are needed to investigate this potential link.

Whether *B. dorei* plays a role in causing T1D autoimmunity in these children or is simply a consequence of emerging autoimmunity, overall health, or yet another environmental factor is unknown. If *B. dorei* is involved in he development of autoimmunity, likely routes include disruption of the epithelial layer and/or manipulation of immune system development. If this is the case, can high levels of *B. dorei* gut colonization be prevented? *Bacteroides* strains are resistant to antibiotics commonly used in pediatrics such as amoxicillin. The *B. dorei* genome sequenced from these stool samples possesses over 50 genes annotated as antibiotic resistance determinants (Leonard et al., 2014). However, two antibiotics, doripenem and tigecycline, are both injected to treat intra-abdominal infections caused by *B. dorei* (Brook et al., 2013). Whether they would be useful in reducing *B. dorei* gut colonization through oral administration is unclear. Alternatively, *Bifidobacterium* strains decrease the population of *B. vulgatus* in the gut (Shiba et al., 2003) and ameliorate gut inflammation by reducing *B. vulgatus* gut populations of gnotobiotic mice (Setoyama et al., 2003).

Another possibility is that a diet change could reduce the population size of *B. dorei*. Previous work has shown that the proportion of children with *B*. *vulgatus* or *B. dorei* in the gut declines when a gluten-free diet is introduced after diagnosis of celiac disease (Sanchez et al., 2010). In addition, the gut microbiome of children at high risk for celiac disease are more likely to include *B. dorei* or *B. vulgatus* than the microbiomes of low risk children (Sanchez et al., 2011). In addition, high protein and fat diets are correlated with high *Bacteroides* populations (Wu et al., 2011).

Results presented here show that the microbiome differs within a window of several months preceding the appearance of the first anti-islet autoantibodies. As such, even if *B. dorei* is not the cause of T1D autoimmunity, its abundance in the gut may be useful as a diagnostic in high-risk groups, such as first-degree relatives, at 6–8 months of age, prior to the appearance of a single autoantibody. At present, infants are not commonly screened for their HLA genotype at birth. Hence, a child's high genetic risk for T1D is only easily determined by the parents if the child has a first degree relative with the disease. As our knowledge of microbiome associations with T1D expands, gut microbiome testing through non-invasive stool analysis might be recommended in the future for infants with a first degree relative affected by T1D.

Type 1 diabetes is a complex disease and is thought to be a cluster of similar diseases with different etiologies (Knip et al., 2005). Studying multiple forms of a disease as a whole may mask differences specific to a single sub-type and fail to discover novel associations. For example, in the BABYDIET study (Endesfelder et al., 2014), which examined the development microbiomes of children from across Germany, no associations were found. In this study, all subjects were born in the same hospital in Turku, Finland and remained in Turku and surrounding areas for the study's duration. As a result, many factors are very similar for all Turku children across this cohort such as climate, health standards and practices, quality of drinking water, exposure to environmental pollutants, diet, and vitamin D levels from sun exposure. Controlling of all of these environmental factors may increase the likelihood that subjects who develop type-1 diabetes have the same form of the disease with the same etiology. Thus, studying large cohorts from one city or small geographic region is particularly valuable.

*Bacteroides dorei* may or may not be associated with T1D autoimmunity in other disease forms, which are more prevalent in other locations. Alternatively, and perhaps more likely, the key to understanding the role of the microbiome in T1D may not be the identification of a specific bacterium, but a set of specific bacterial genes related to gut health. It just so happens, that in this one city, Turku, Finland, those functions may largely be associated with one bacterial species.

### Conflict of interest statement

The authors declare that the research was conducted in the absence of any commercial or financial relationships that could be construed as a potential conflict of interest.
