# Supplementary material for: Bacteroides dorei dominates gut microbiome prior to autoimmunity in Finnish children at high risk for type 1 diabetes
Source: Front Microbiol. 2014 Dec 10;5:678. doi: 10.3389/fmicb.2014.00678 (PMC4261809; doi:10.3389/fmicb.2014.00678)
Supplement: Supplementary file 11 [file Presentation1.ZIP › Supplementary Methods/Antibiotics.pdf]

Antibiotics

Setup

```
library(ggplot2)
library(dplyr) #sometimes dplyr don't work
library(magrittr)
library(ggthemr)
library(knitr)
library(phyloseq)
ggthemr('fresh')
```

```
## Warning: New theme missing the following elements: panel.margin.x,
## panel.margin.y
```

```
source('lib/load_dipp.R')

# shutup, ggplot
opts_chunk$set(warning=FALSE, message=FALSE)
```

Load Data

```
# load csv files
antibiotics <- read.csv('../data/antibiotics-courses.csv')
subjects <- load_dipp_subject_data('../data/dipp-subjects.csv')
categories <- read.csv('../data/antibiotics-categories.csv')

# add subject data to antibiotics table
merged <- merge(antibiotics, subjects, by.x='dipp_person', by.y='dipp_person')
merged <- merge(merged, categories, by.x='type', by.y='antibiotic', all.x=T)
```

Antibiotic Categories

I looked up the antibiotics in Wikipedia and made a table of their categories. There are multiple levels of category so I took the most broad.

```
categories
```

```
##                           antibiotic          category
## 1                            Unknown           Unknown
## 2                               None              None
## 3                        Amoxycillin       beta-lactam
## 4                       Azithromycin azalide/macrolide
## 5                           Cefactor      cephalosporin
## 6                          Cephalexin     cephalosporin
## 7                      Cephalosporin     cephalosporin
## 8                           Cefaclor     cephalosporin
## 9                     Clarithromycin         macrolide
## 10                        Gentamicin    aminoglyciside
## 11        Phenoxymethylpenicillin              penam
## 12                       Trimethoprim        antifolate
## 13 Trimethoprim-sulfamethoxazole        antifolate
## 14                   Chloramphenicol       tetracyclin
## 15                      Fucidic acid           fusidic
```

Any missing categories?

```
types = unique(merged$type)
types[!types %in% categories$antibiotic]
```

```
## [1] Unknown prophylactic
## 15 Levels: Amoxycillin Azithromycin Cefaclor Cephalexin ... Unknown prophylactic
```

Munging

Ignore this step. I'm just fixing the data so R is happy.

```
merged <- within(merged, {
  ab.start <- as.numeric(as.character(ab.start))
  ab.stop <- as.numeric(as.character(ab.stop))
  duration = (ab.stop - ab.start)
})

# only care about Turku, Finland right now
merged <- subset(merged, site == 'Turku')

# remove 'None'. We don't need this in the table to do statistics.
merged <- subset(merged, type != 'None')

# only care about pre seroconversion
```

Total courses

Compare total courses (prescriptions) of antibiotics between cases and controls.

```
ggplot(merged,
       aes(x=seroconverted)) +
geom_bar()
```

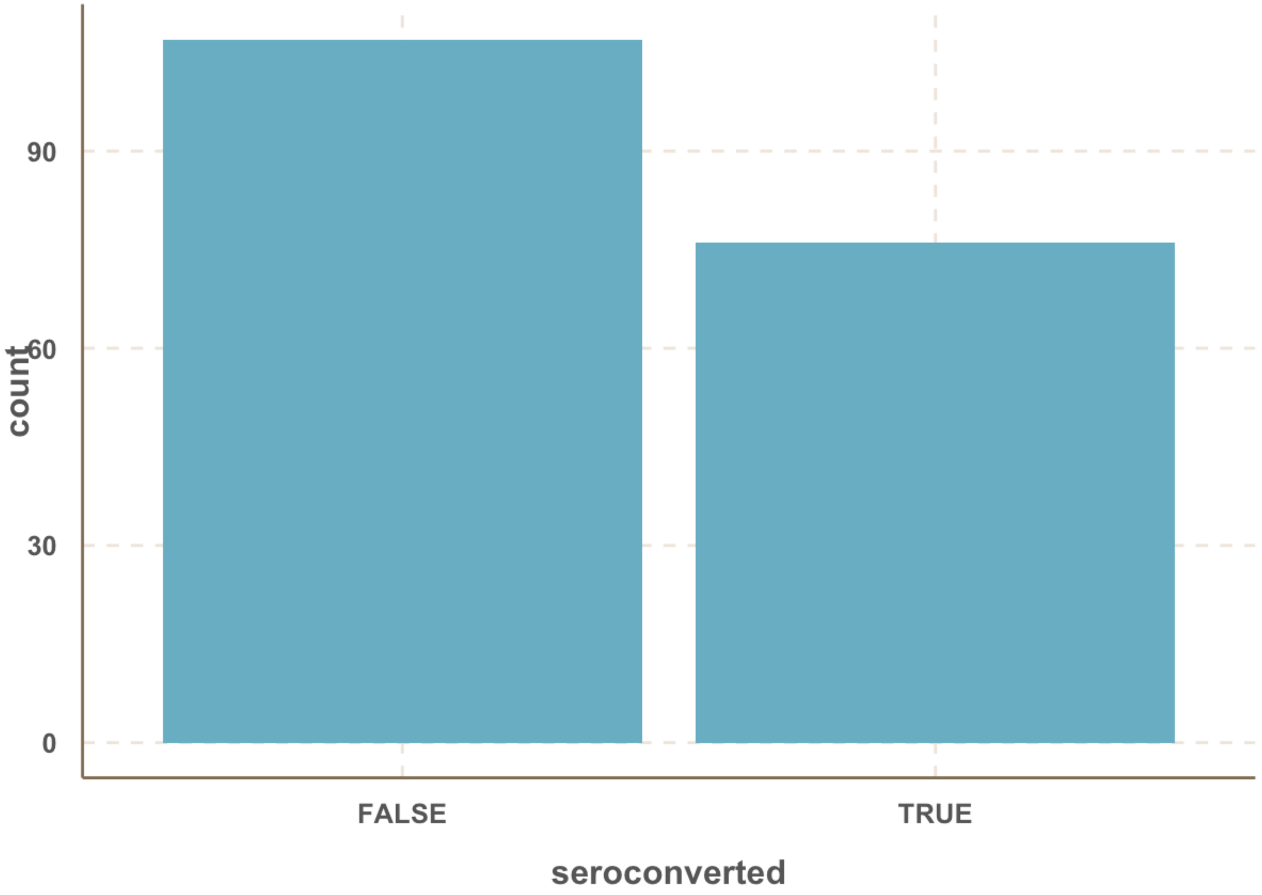

Total Courses (by antibiotic)

Total courses of antibiotic by type between cases and controls.

```
ggplot(merged,
       aes(x=type,
           fill=seroconverted)) +
geom_bar(position='dodge') +
coord_flip()
```

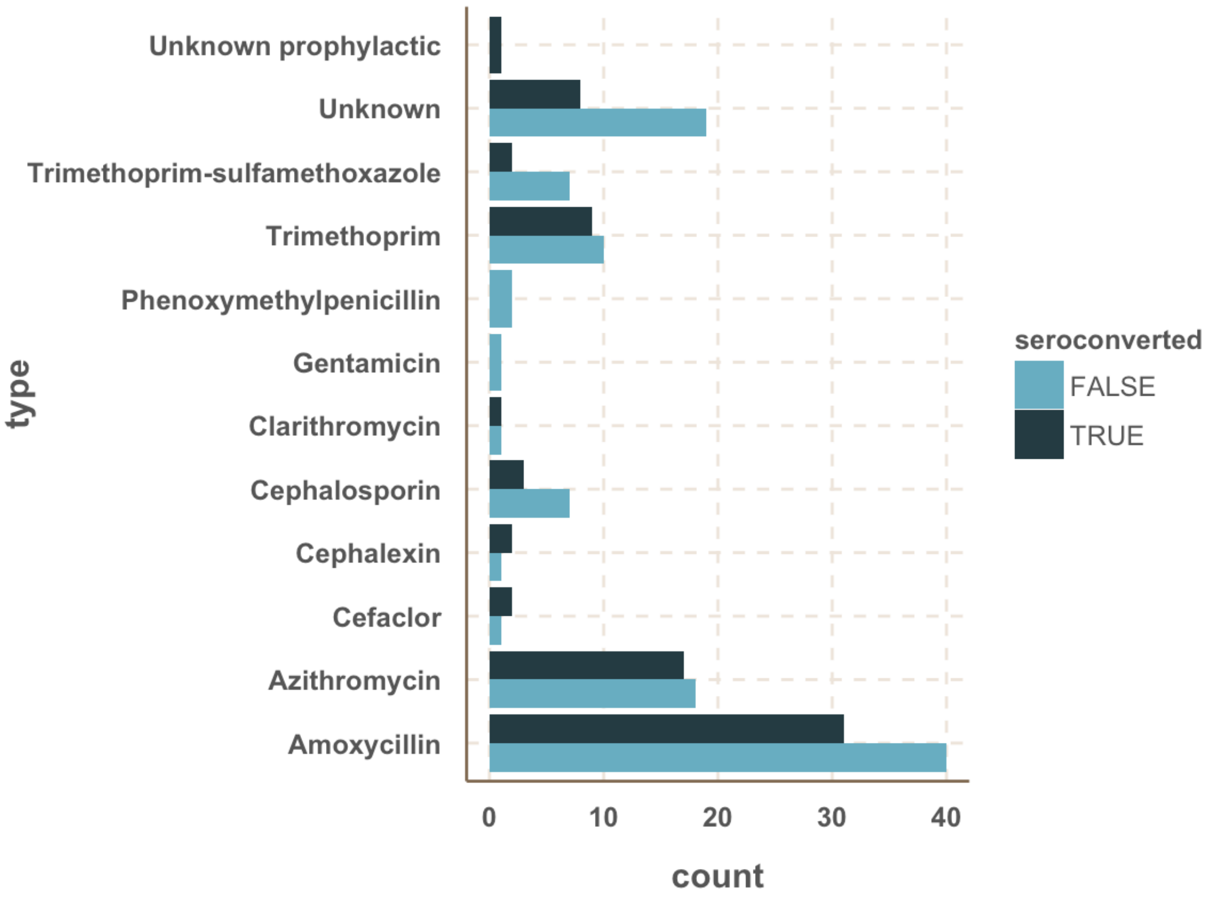

Total Courses (categories)

Total courses of antibiotic by category between cases and controls.

```
ggplot(merged,
       aes(x=category,
           fill=seroconverted)) +
geom_bar(position='dodge') +
coord_flip()
```

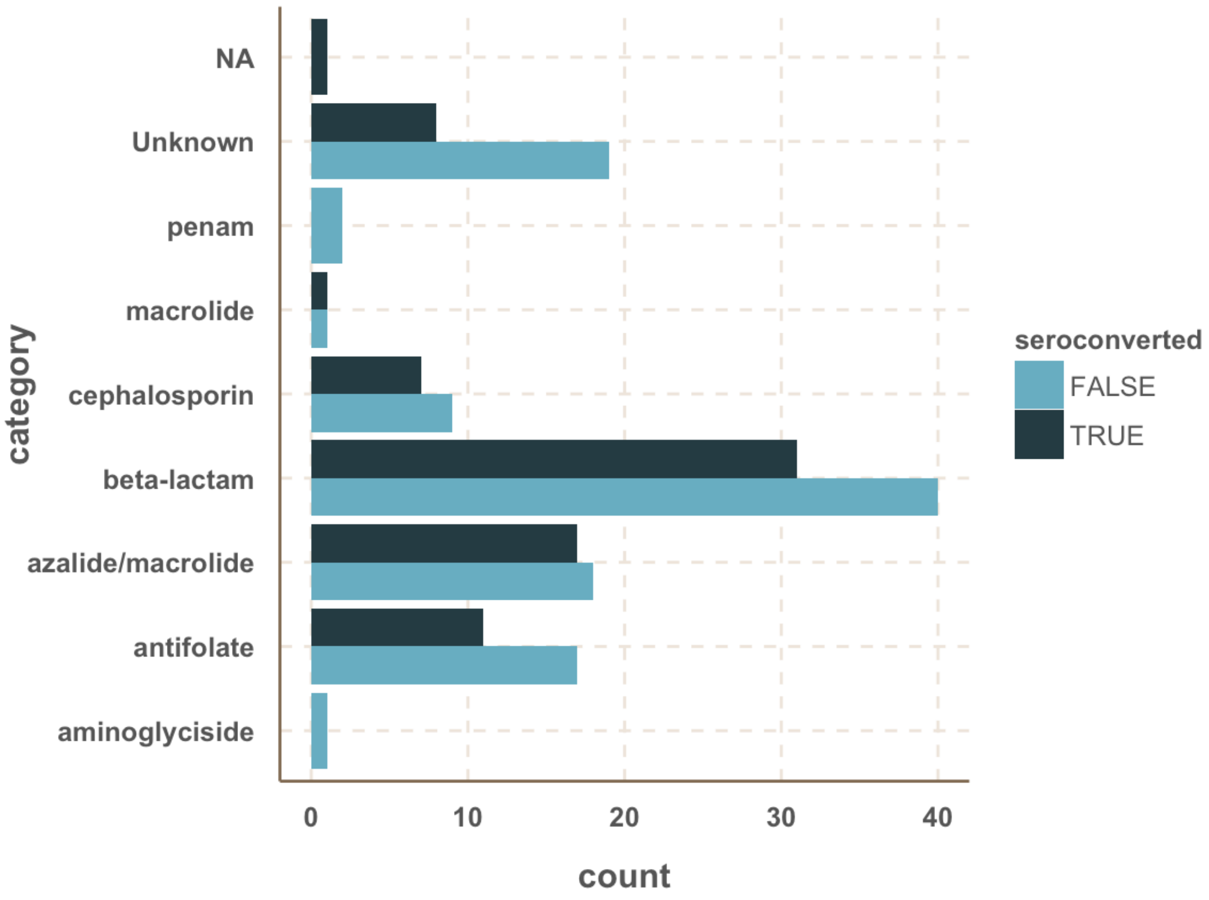

Test for differences (all samples)

What's different between cases and controls?

Total Courses

Did total courses of antibiotics differ between cases and controls?

```
# this function will perform two chi-square tests: the first testing
# if cases an controls received different prescriptions for antibiotics ever
# and the second before a specific month cutoff (default = 7.4 months).

test_antibiotic <- function(df, name,  month_cutoff = 7.4) {
  anti <- subset(df, type == name)
  t1 <- chisq.test(table(anti$seroconverted),
             simulate.p.value=TRUE)
  p1 <- sprintf('%.3f', t1$p.value)

  anti2 <- subset(anti, ab.start < (month_cutoff * 30))

  if (nrow(anti2) != 0) {
    t2 <- chisq.test(table(anti2$seroconverted),
                  simulate.p.value=TRUE)
    p2 <- sprintf('%.3f', t2$p.value)
  } else {
    p2 <- NA
  }

  paste('all: ', p1 , 'before', month_cutoff, ' months:', p2 )
}
```

```
merged$seroconverted <- as.factor(merged$seroconverted)
for (ab in unique(merged$type)) {
  print(ab)
  print(test_antibiotic(merged, ab))
}
```

```
## [1] "Amoxycillin"
## [1] "all:  0.352 before 7.4  months: 0.722"
## [1] "Azithromycin"
## [1] "all:  1.000 before 7.4  months: 1.000"
## [1] "Cefaclor"
## [1] "all:  1.000 before 7.4  months: 1.000"
## [1] "Cephalexin"
## [1] "all:  1.000 before 7.4  months: 1.000"
## [1] "Cephalosporin"
## [1] "all:  0.369 before 7.4  months: 0.489"
## [1] "Clarithromycin"
## [1] "all:  1.000 before 7.4  months: NA"
## [1] "Gentamicin"
## [1] "all:  1.000 before 7.4  months: 1.000"
## [1] "Phenoxymethylpenicillin"
## [1] "all:  0.503 before 7.4  months: NA"
## [1] "Trimethoprim"
## [1] "all:  1.000 before 7.4  months: 1.000"
## [1] "Trimethoprim-sulfamethoxazole"
## [1] "all:  0.180 before 7.4  months: 0.506"
## [1] "Unknown"
## [1] "all:  0.054 before 7.4  months: 0.073"
## [1] "Unknown prophylactic"
## [1] "all:  1.000 before 7.4  months: NA"
```

Age of First Antibiotics

```
ggplot(first,
      aes(x=seroconverted,
          y=ab.start)) +
geom_boxplot()
```

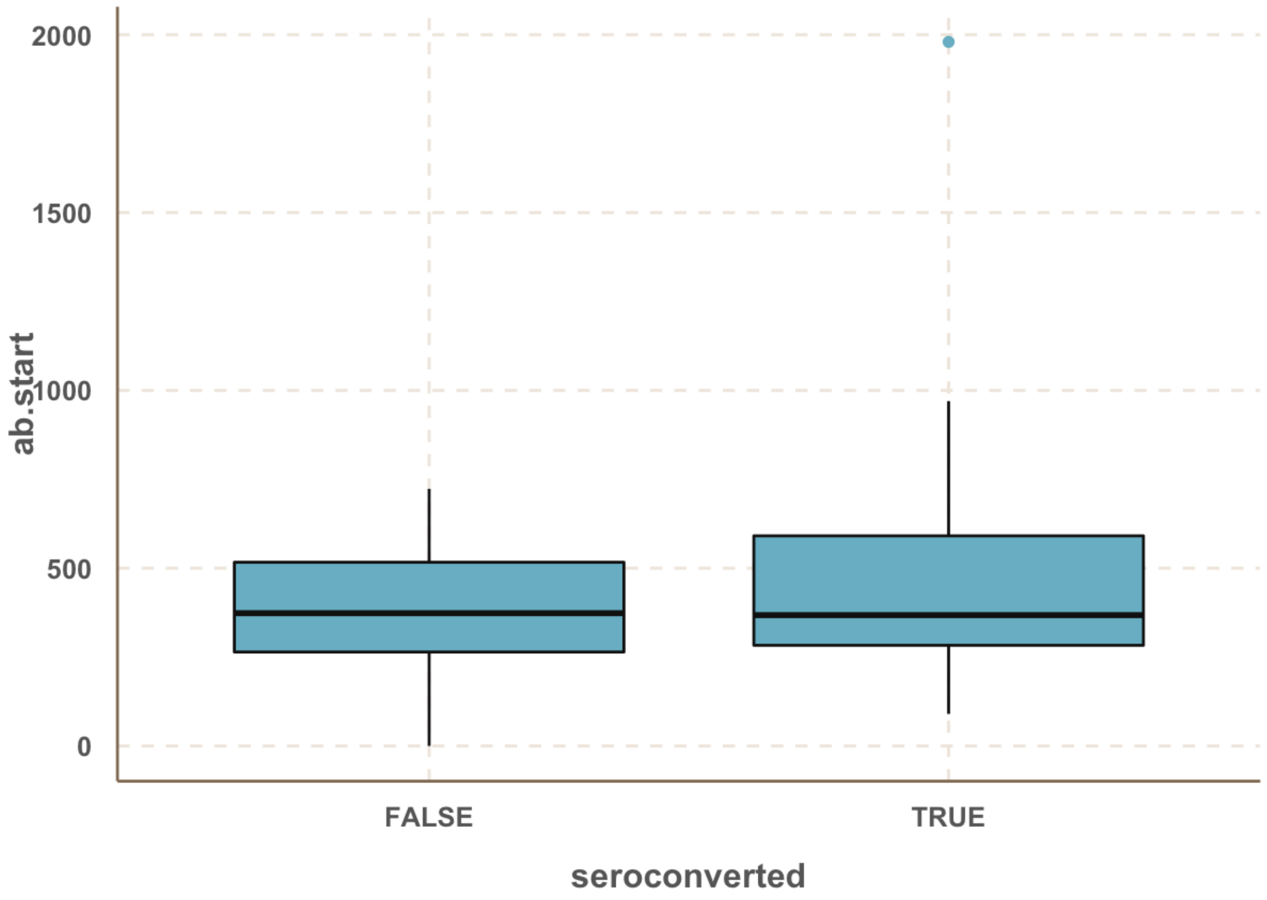


```
wilcox.test(ab.start ~ seroconverted,
       data=merged)
```

```
##
##  Wilcoxon rank sum test with continuity correction
##
## data:  ab.start by seroconverted
## W = 3588, p-value = 0.1763
## alternative hypothesis: true location shift is not equal to 0
```
