# Supplementary material for: Bacteroides dorei dominates gut microbiome prior to autoimmunity in Finnish children at high risk for type 1 diabetes
Source: Front Microbiol. 2014 Dec 10;5:678. doi: 10.3389/fmicb.2014.00678 (PMC4261809; doi:10.3389/fmicb.2014.00678)
Supplement: Supplementary file 11 [file Presentation1.ZIP › Supplementary Methods/Bacteroides qPCR.pdf]

Predict *B. dorei* cells per g. stool

I'm going to attemp to extrapolated the number of *B. dorei* 16S rRNA genes from qPCR done using *Bacteroides*-specific 16S rRNA primers, 16S rRNA sequencing data, mass of stool samples in grams and DNA concentration within those stool samples.

Build model to extrapolate 16S rRNA copy number

Load data

- All DIPP data
- Predicted *B. dorei* relative abundance from MiSeq.
- qPCR on *Bacteroides*.

```
# Original HiSeq Data
load('dipp.Rdata')
dipp.all <- dipp

print(dipp.all)
```

```
## phyloseq-class experiment-level object
## otu_table()   OTU Table:         [ 3982 taxa and 1064 samples ]
## sample_data() Sample Data:       [ 1064 samples by 74 sample variables ]
## tax_table()   Taxonomy Table:    [ 3982 taxa by 9 taxonomic ranks ]
```

```
# Predicted B. dorei relative abundance from MiSeq data
load('dipp-predicted.Rdata')
dipp.predicted <- dipp

# qPCR Data
qpcr <- read.csv('../data/qpcr-data/bacteroides-qPCR.csv')
qpcr$sample_id <- as.character(qpcr$sample)
qpcr$copies <- as.numeric(as.character(qpcr$copies))
```

Take the median and standard error of the copy number from the qPCR data.

```
qpcr$sample_id <- as.factor(qpcr$sample_id)
avg <- ddply(qpcr, ~ sample_id, function(x) {
  c(median=median(x$copies), se=se(x$copies))
})
```

Get just the *Bacteroides* from the HiSeq data

Poisson regression requires integer counts so the proportions are multiplied by a largish number and then rounded.

```
bacteroides <- dipp.all %>%
  subset_samples(site == 'Turku') %>%
  subset_samples(sequencer == 'Illumina HiSeq') %>%
```

```
  subset_samples(aa_number != 1) %>%
  transform_sample_counts(function(x) round(10000000*x)) %>%
  subset_taxa(Genus == 'Bacteroides') %>%
  tax_glom(taxrank = 'Genus') %>%
  psmelt()
```

Merge *Bacteroides* relative abundance data with qPCR data

```
merged <- merge(bacteroides, qpcr, by.x='sample_id', by.y='sample_id')
merged$median <- merged$copies
```

Also merge *B. dorei* relative abundance

```
# Load B. dorei predicted relative abundance
load('dipp-predicted.Rdata')
dorei <- dipp %>%
  subset_taxa(Species == 'dorei') %>%
  psmelt()

# four hour bug right there...
rownames(dorei) <- dorei$Sample
rownames(bacteroides) <- bacteroides$Sample

# Add B. dorei abundance to Bacteroides data.frame
common <- intersect(rownames(bacteroides), rownames(dorei))
bacteroides <- bacteroides[common,]
dorei <- dorei[common,]
bacteroides$dorei <- dorei$Abundance
```

Correlate Relative Abundance with Absolute (qPCR) Abundance

Extrapolate *Bacteroides* copy number based on # of reads

Fit Regression with GLM (Poisson and Log Family)

```
# make sure median is an integer b/c of Poisson
merged$median <- as.integer(merged$median)

# remove NAs
merged <- merged[complete.cases(merged$median),]

# fit LM
m0 <- glm(median ~ Abundance , data=merged, family=poisson(link='log'))
```

```
bacteroides$predicted <- predict(m0, newdata=bacteroides, type='response')

# Plot
```

```r
ggplot(bacteroides,
       aes(x=Abundance,
           y=predicted)) +
geom_point() +
geom_point(aes(x=Abundance,
               y=median),
           data=merged,
           color='red')
```

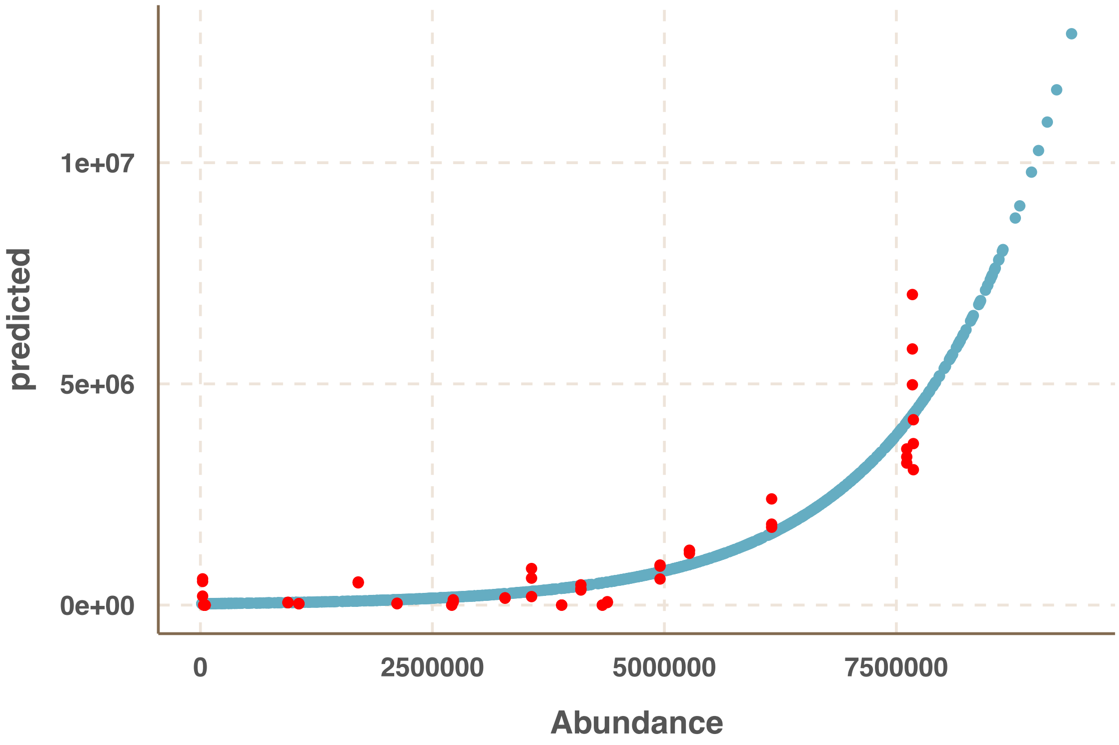

(Red dots are original qPCR data points)

Estimate cells of *Bacteroides* per gram of stool

To do this I will use the following data

- (Predicted) 16S rRNA copy number [copies]
- Dilution factor for qPCR `(1/10)` [unitless]
- DNA concentration in DNA extraction from stool nanodrop [ng/uL]
- Volume of DNA extracted from X grams of stool [uL]
- Mass of stool [g]
- *Bacteroides* and *B. dorei* 16S rRNA copy number [copies/cell].

If you do the stoichiometry, you should get cells/grams of stool as your final units:

```
(16S copies / 10-ng DNA) * (10/1) * (x ng/uL DNA) * (50 uL/1 g stool) * (6 Bacteroides
16S rRNA / 1 genome) = [Bacteroides cells / gram of stool]
```

First calculate total 16S rRNA copies, then calculate the cell count for *Bacteroides* and *B. dorei* separately.

Calculate *Bacteroides* cells per gram of stool

```
bacteroides <- within(bacteroides, {
    copies <- (predicted) * (10/1) * (nanodrop) * (50) * (1/stool_mass_g)
})
```

Calculate the % *B. dorei* cells from their proportion of the *Bacteroides*

Estimate *B. dorei* 16S rRNA copy number

```
ggplot(bacteroides,
       aes(x=age_at_sampling/30,
           y=copies/1e9,
           fill=seroconverted,
           color=seroconverted,
           linetype=seroconverted)) +
geom_smooth() +
scale_x_continuous(breaks=seq(0, 48, by=3), limits=c(3, 24)) +
ggtitle('Bacteroides') +
ylab('Estimated 16S rRNA copies (billions)') +
xlab('Age at Sampling (months)') +
ggplot.theme + ggplot.pal.scale + ggplot.color
```

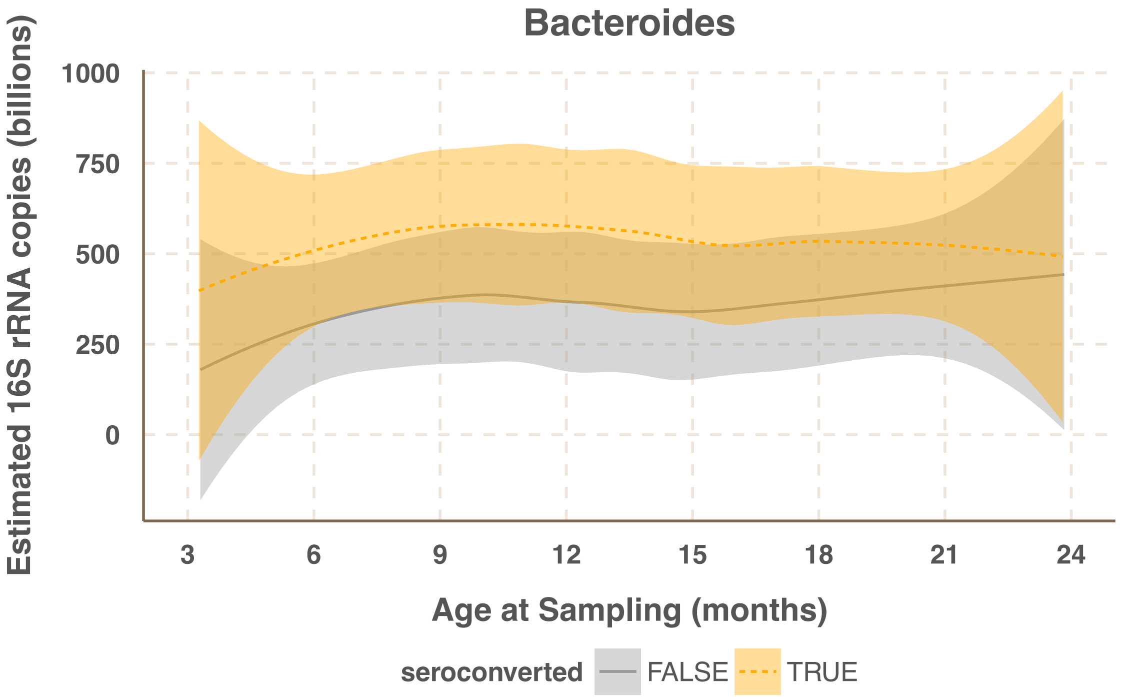


```
ggplot(bacteroides,
       aes(x=age_at_sampling/30,
           y=(copies/6)/1e9,
           fill=seroconverted,
```

```
            color=seroconverted,
            linetype=seroconverted)) +
geom_smooth() +
scale_x_continuous(breaks=seq(0, 48, by=3), limits=c(3, 24)) +
geom_hline(y=1e11/1e9) +
ggtitle('Bacteroides') +
ylab('Billions of cells per gram of stool') +
xlab('Age at Sampling (months)') +
ggplot.theme + ggplot.pal.scale + ggplot.color
```

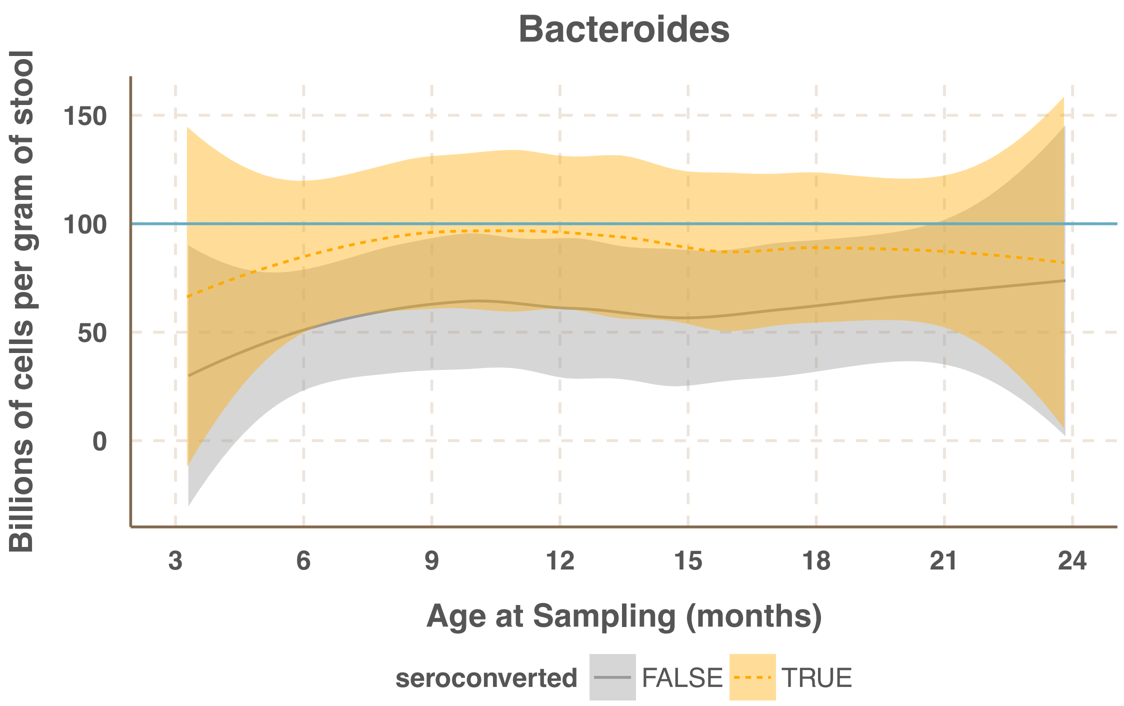


```
ggplot(bacteroides,
       aes(x=age_at_sampling/30,
           y=copies * dorei/1e9,
           fill=seroconverted,
           color=seroconverted,
           linetype=seroconverted)) +
geom_smooth() +
scale_x_continuous(breaks=seq(0, 48, by=3), limits=c(3, 24)) +
ggtitle('Bacteroides dorei') +
ylab('Billions of cells per gram of stool') +
xlab('Age at Sampling (months)') +
ggplot.theme + ggplot.pal.scale + ggplot.color
```

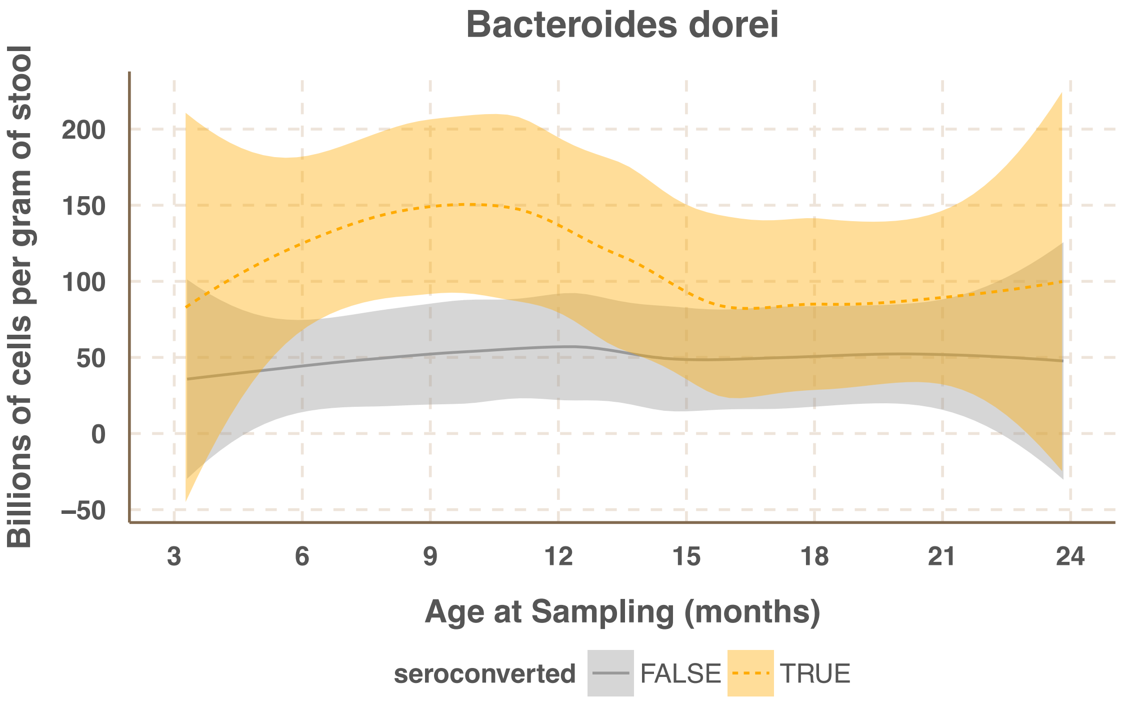
Bacteroides dorei
Billions of cells per gram of stool
200
150
100
50
0
−50
3
6
9
12
15
18
21
24
Age at Sampling (months)
seroconverted
FALSE
TRUE
