# Supplementary material for: Bacteroides dorei dominates gut microbiome prior to autoimmunity in Finnish children at high risk for type 1 diabetes
Source: Front Microbiol. 2014 Dec 10;5:678. doi: 10.3389/fmicb.2014.00678 (PMC4261809; doi:10.3389/fmicb.2014.00678)
Supplement: Supplementary file 11 [file Presentation1.ZIP › Supplementary Methods/Confounders.pdf]

Confounders

```
library(ggplot2)
library(magrittr)
library(dplyr)
```

```
source('lib/load_dipp.R')
dat <- fix_subject_columns(read.csv('../data/dipp-subjects.csv'))
```

Mode of Delivery

Vaginal vs. C-section

```
chisq.test(table(dat$vaginal_delivery,
      dat$seroconverted))
```

```
##
##  Pearson's Chi-squared test with Yates' continuity correction
##
## data:  table(dat$vaginal_delivery, dat$seroconverted)
## X-squared = 0.4739, df = 1, p-value = 0.4912
```

Breast Feeding

breast feeding (any)

```
x <- subset(dat, Breast_feeding_any != '') # remove blanks
chisq.test(table(dat$Breast_feeding_any,
                 dat$seroconverted))
```

```
## Warning: Chi-squared approximation may be incorrect
```

```
##
##  Pearson's Chi-squared test
##
## data:  table(dat$Breast_feeding_any, dat$seroconverted)
## X-squared = 1.552, df = 2, p-value = 0.4604
```

Breast feeding (duration)

```
wilcox.test(Duration_Breast_Feeding_months ~ seroconverted,
            data=dat)
```

```
##
##  Wilcoxon rank sum test with continuity correction
##
## data:  Duration_Breast_Feeding_months by seroconverted
## W = 1500, p-value = 0.1198
## alternative hypothesis: true location shift is not equal to 0
```

Breast Feeding (exclusive duration)

```r
wilcox.test(Duration_exclusive_breast_feeding_weeks ~ seroconverted,
            data=dat)
```

```
##
##  Wilcoxon rank sum test with continuity correction
##
## data:  Duration_exclusive_breast_feeding_weeks by seroconverted
## W = 1488, p-value = 0.1044
## alternative hypothesis: true location shift is not equal to 0
```

Gender

```r
chisq.test(table(dat$Gender,
                 dat$seroconverted))
```

```
##
##  Pearson's Chi-squared test with Yates' continuity correction
##
## data:  table(dat$Gender, dat$seroconverted)
## X-squared = 0, df = 1, p-value = 1
```

HLA Risk (moderate vs high)

```r
chisq.test(table(dat$risk < 5,
                 dat$seroconverted))
```

```
##
##  Pearson's Chi-squared test with Yates' continuity correction
##
## data:  table(dat$risk < 5, dat$seroconverted)
## X-squared = 0.0519, df = 1, p-value = 0.8198
```
