# Supplementary material for: Bacteroides dorei dominates gut microbiome prior to autoimmunity in Finnish children at high risk for type 1 diabetes
Source: Front Microbiol. 2014 Dec 10;5:678. doi: 10.3389/fmicb.2014.00678 (PMC4261809; doi:10.3389/fmicb.2014.00678)
Supplement: Supplementary file 11 [file Presentation1.ZIP › Supplementary Methods/MiSeq Analysis.pdf]

MiSeq Analysis

Description

The goal is to use MiSeq data which can distinguish *B. dorei* from *B. vulgatus* to estimate the relative abundance of *B. dorei* and *B. vulgatus* in the samples sequenced with shorter reads HiSeq based on the combined relative abundance of the *B. dorei/vulgatus* cluster.

Load Data

```r
system('make dipp.Rdata')
load('dipp.Rdata')
hiseq <- dipp

system('make dipp-miseq.Rdata')
load('dipp-miseq.Rdata')
miseq <- dipp
```

MiSeq Summary

- There are 93 samples.
- 38 subjects.
- 51 case samples.
- 42 control samples.
- 19 case subjects
- 19 case subjects

HiSeq Summary

- There are 1064 samples.
- 81 subjects.
- 584 case samples.
- 480 control samples.
- 34 case subjects
- 47 case subjects

Data Munging

There are 93 samples in common.

B. dorei relative abundance across age in the MiSeq Data.

You don't see the *B. dorei* spike in this graph because we only sequenced 93 samples using MiSeq.

```
miseq %>%
  subset_samples(site == 'Turku') %>%
  subset_taxa(Species %in% c('vulgatus', 'dorei')) %>%
  tax_glom(taxrank = 'Species') %>%
  psmelt() %>%
  ggplot(aes(x=age_at_sampling,
             y=Abundance,
             color=seroconverted)) +
  geom_smooth() +
  facet_wrap(~Species) +
  xlim(100, 600)
```

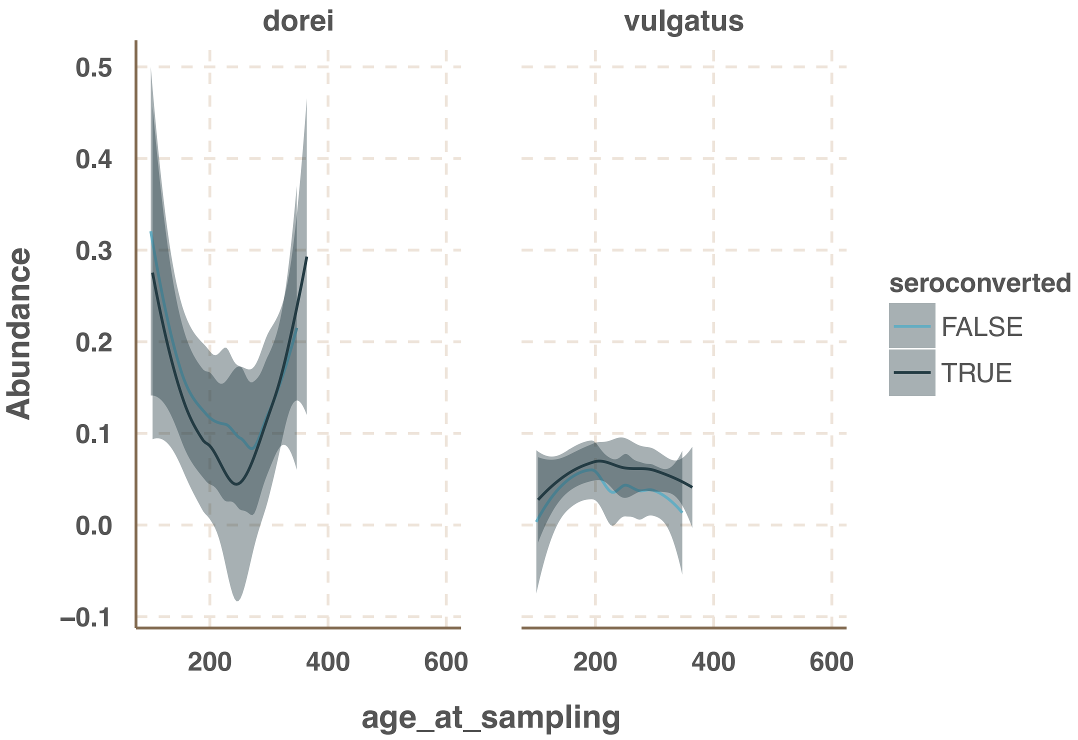

MiSeq/HiSeq Comparison

HiSeq (Combined) vs. MiSeq (*B. dorei*)

This is a plot of *B. dorei/vulgatus* relative abundance from HiSeq samples and *B. dorei* relative abundance from MiSeq data. We will be fitting a linear regression on this data to predict *B. dorei* relative abundance from HiSeq data relative abundance from HiSeq data.

A 40% relative abundance from HiSeq corresponds to about a 20% relative abundance on MiSeq. Also, there are a lot of outliers and the fit ins't that good. This is okay though because we will use robust linear regressions!

```
ggplot(vs,
       aes(x=hiseq,
           y=miseq.dorei,
           color=seroconverted)) +
geom_point() +
```

```r
scale_y_continuous(labels=percent) +
scale_x_continuous(labels=percent)
```

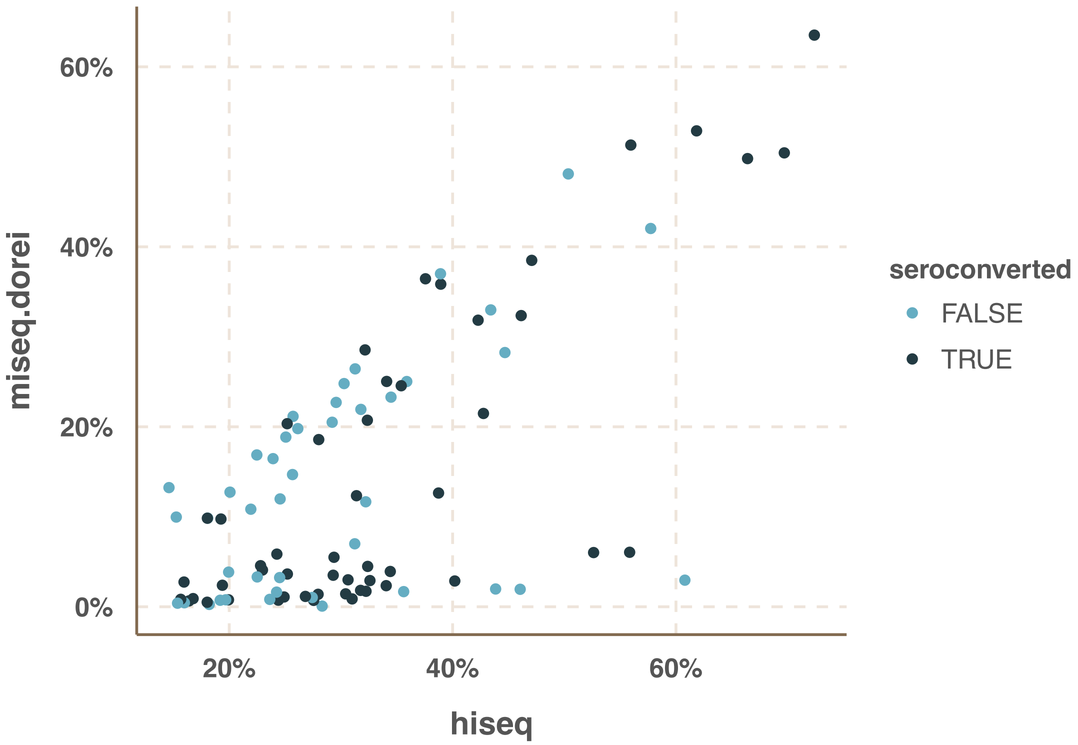

Predict *B. dorei* and *B. vulgatus* across all HiSeq samples

Fit linear regressions with hiseq rel. abund. as predictor and miseq *B. dorei* and *B. vulgatus* as response

note: the +0 term is to set the y-intercept at 0. Otherwise, # the linear model will try to estimate the y-intercept and as a result # we will get negative predicted relative abundances which doesn't make sense

We're using a special robust linear regression with weights proportional to relative abundance b/c the low relative abundance samples have the highest variance. This is probably because the relative abundance is reaching the limit of quantification (LOQ) of the Illumina sequencer.

```r
# without eiths
m.dorei.0 <- lm(miseq.dorei ~ hiseq+0, data=vs)

range01 <- function(x){(x-min(x))/(max(x)-min(x))}
# with weights
m.dorei <- lm(miseq.dorei ~ hiseq+0, data=vs, weights=range01(vs$miseq.dorei))
m.vulgatus <- lm(miseq.vulgatus ~ hiseq+0, data=vs, weights=range01(vs$miseq.vulgatus))
```

Perform a likelihood ratio test to see if the weighted model is better than the unweighted model.

```r
r <- anova(m.dorei.0, m.dorei, test='LRT')
print(r)
```

```
## Analysis of Variance Table
```

```
##
## Model 1: miseq.dorei ~ hiseq + 0
## Model 2: miseq.dorei ~ hiseq + 0
##   Res.Df   RSS Df Sum of Sq Pr(>Chi)
## 1     92 1.307
## 2     91 0.138  1      1.17   <2e-16 ***
## ---
## Signif. codes:  0 '***' 0.001 '**' 0.01 '*' 0.05 '.' 0.1 ' ' 1
```

p-value: 2.6998 × 10-169 so we use the weighted model.

```r
anova(m.dorei)
```

```
## Analysis of Variance Table
##
## Response: miseq.dorei
##           Df Sum Sq Mean Sq F value Pr(>F)
## hiseq      1  2.282   2.282    1502 <2e-16 ***
## Residuals 91  0.138   0.002
## ---
## Signif. codes:  0 '***' 0.001 '**' 0.01 '*' 0.05 '.' 0.1 ' ' 1
```

```r
dfh$hiseq <- dfh$Abundance

dfh$predicted.dorei <- predict(m.dorei, newdata=dfh, type='response')
dfh$predicted.vulgatus <- predict(m.vulgatus, newdata=dfh, type='response')

ggplot(dfh,
       aes(x=hiseq,
           y=predicted.dorei)) +
geom_point() +
scale_x_continuous(labels=percent) +
scale_y_continuous(labels=percent)
```

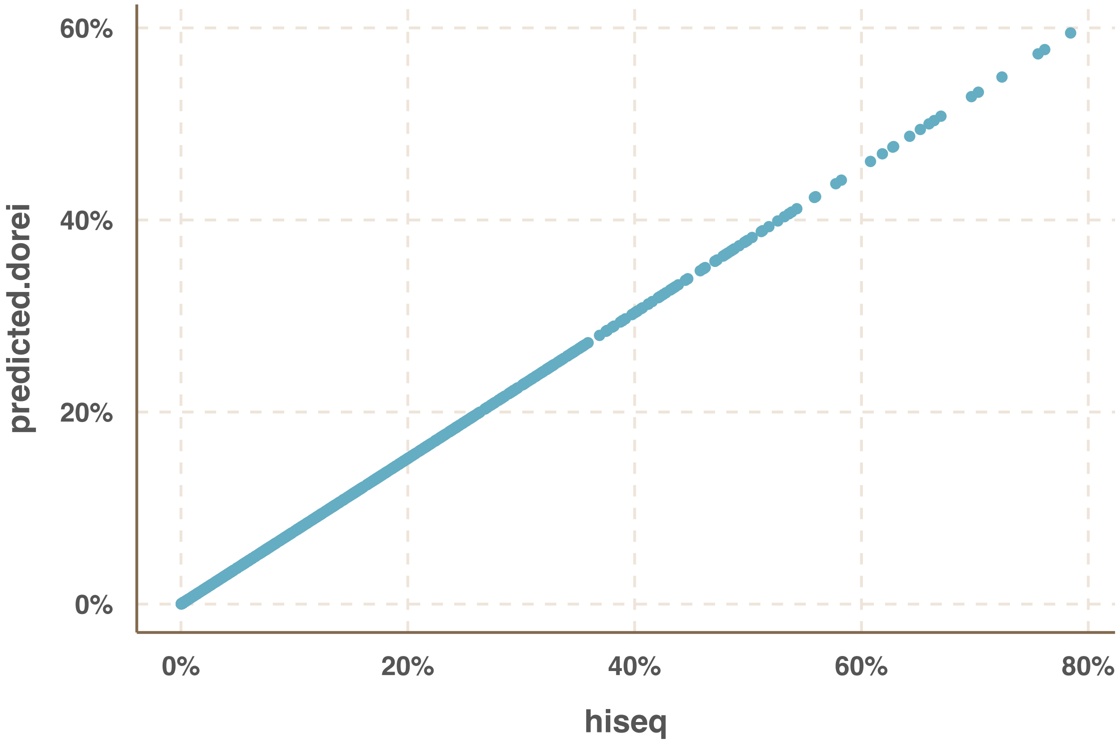


```
ggplot(dfh,
       aes(x=hiseq,
           y=predicted.vulgatus)) +
geom_point() +
scale_x_continuous(labels=percent) +
scale_y_continuous(labels=percent)
```

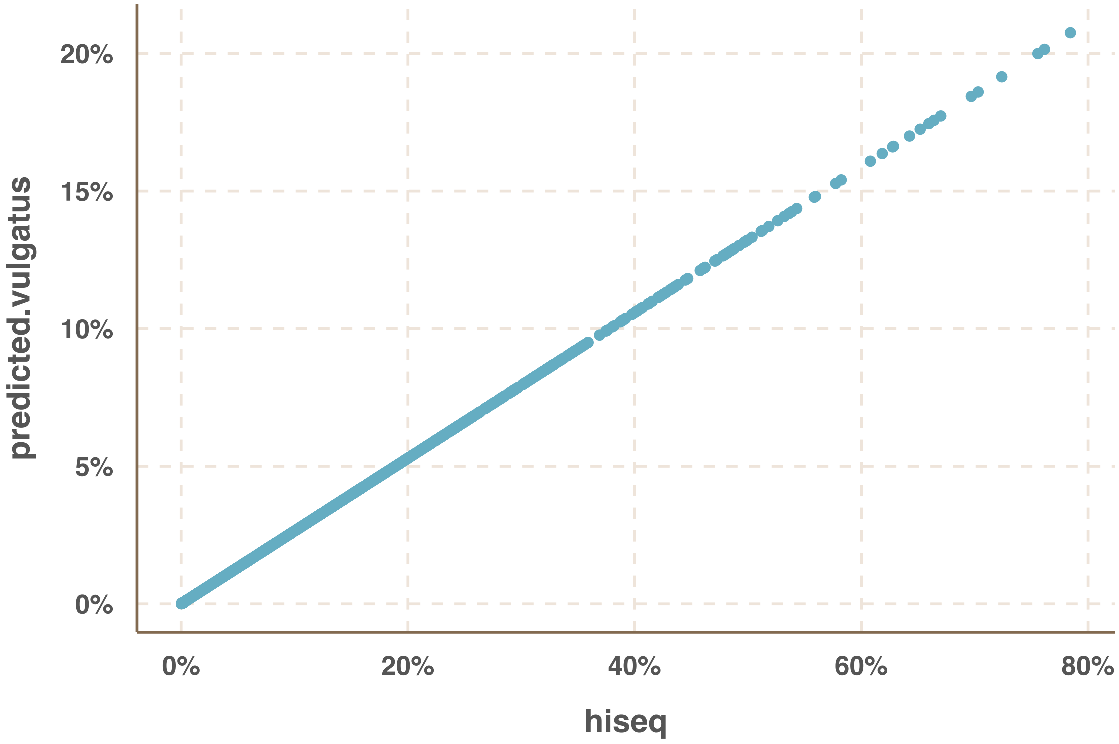

Predicted relative abundance of *B. dorei* and *B. vulgatus*

```
p1 <- ggplot(dfh,
       aes(x=age_at_sampling,
           y=predicted.dorei,
           color=seroconverted)) +
geom_smooth() +
xlim(100, 600) +
scale_y_continuous(labels=percent) +
ggtitle('Estimated B. dorei') +
ylab('Relative Abundance') +
xlab('Age At Sampling')
```

```
p2 <- ggplot(dfh,
       aes(x=age_at_sampling,
           y=predicted.vulgatus,
           color=seroconverted)) +
geom_smooth() +
xlim(100, 600) +
scale_y_continuous(labels=percent) +
ggtitle('Estimated B. vulgatus') +
ylab('Relative Abundance') +
xlab('Age At Sampling')
```

Combined B. dorei and B. vulgatus Does it match original HiSeq data?

```
ggplot(dfh,
        aes(x=age_at_sampling,
            y=predicted.dorei + predicted.vulgatus,
            color=seroconverted)) +
geom_smooth() +
xlim(100, 600) +
scale_y_continuous(labels=percent) +
ggtitle('MiSeq')
```

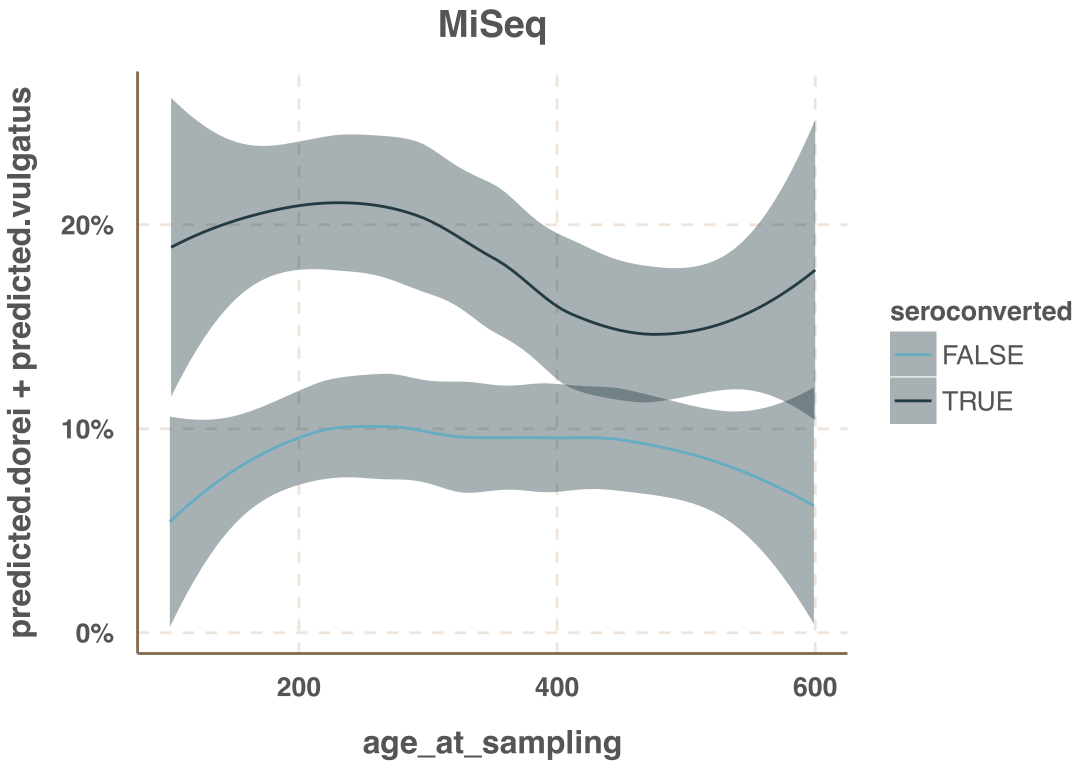


```
hiseq %>%
  subset_samples(site == 'Turku') %>%
  subset_taxa(Species == 'dorei-vulgatus') %>%
  tax_glom(taxrank = 'Species') %>%
  psmelt() %>%
  ggplot(aes(x=age_at_sampling,
             y=Abundance,
             color=seroconverted)) +
  geom_smooth() +
  facet_wrap(~Species) +
  xlim(100, 600) +
  scale_y_continuous(label=percent) +
  ggtitle('HiSeq')
```

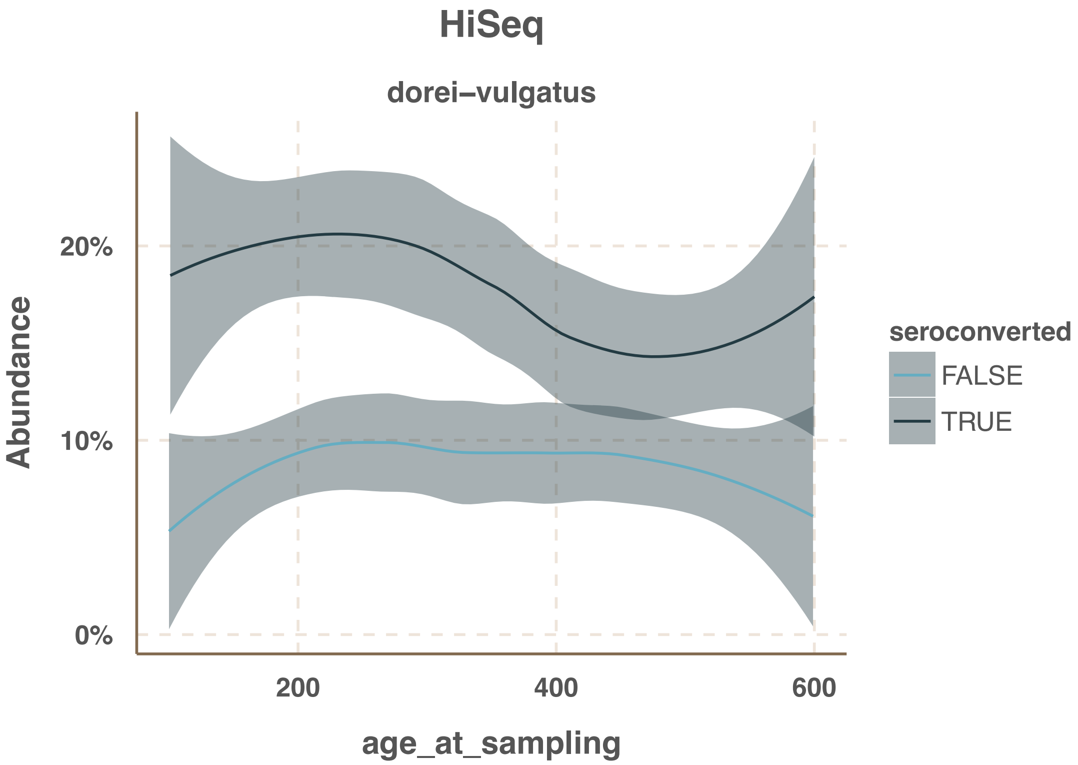

The predicted *B. dorei* and *B. vulgatus* both add up to the combined *B. dorei/vulgatus* from the MiSeq data.

```
# write predicted B. dorei and B. vulgatus OTU tables for use in the GLM script.
x <- data.frame(`3989856`=dfh$predicted.dorei,
                `4399495`=dfh$predicted.vulgatus,
                check.names=F)

rownames(x) <- dfh$Sample

write.csv(x, file='../data/otu-tables/predicted-dorei-vulgatus.csv')
```
