# Supplementary material for: Bacteroides dorei dominates gut microbiome prior to autoimmunity in Finnish children at high risk for type 1 diabetes
Source: Front Microbiol. 2014 Dec 10;5:678. doi: 10.3389/fmicb.2014.00678 (PMC4261809; doi:10.3389/fmicb.2014.00678)
Supplement: Supplementary file 11 [file Presentation1.ZIP › Supplementary Methods/Technical Sequencing Replicates.pdf]

Analysis of Technical Replicates

The goal of this statistical document is to estimate the limit of quantification for measuring relative abundance of bacterial taxa through 16S rRNA sequencing using the Illumina MiSeq and HiSeq 1000 instruments.

Technical replicates were used to measure variance due to experimental conditions:

- Primers
- PCR amplification
- DNA Extraction
- Sequencing Lane
- Sequencing technology
- Sequencing run

```
# cache by default
knitr::opts_chunk$set(cache=TRUE)
# shut up ggplot2
knitr::opts_chunk$set(warning=FALSE, message=FALSE)
```

```
library(phyloseq)
library(ggplot2)
library(plyr)
library(magrittr)
library(ggthemr)
ggthemr('dust')
```

Load DIPP experiment.

```
load('dipp-tech-reps.Rdata')
dipp
```

```
## phyloseq-class experiment-level object
## otu_table()   OTU Table:         [ 3982 taxa and 1327 samples ]
## sample_data() Sample Data:       [ 1327 samples by 70 sample variables ]
## tax_table()   Taxonomy Table:    [ 3982 taxa by 9 taxonomic ranks ]
```

Plot how many of each type of technical replicate we have:

```
dipp %>%
  subset_samples(TechReps) %>%
  sample_data() %>%
  ddply(~TechRep_type, nrow)
```

```
##   TechRep_type  V1
## 1   extraction  30
## 2         lane 200
## 3          pcr  30
## 4        reseq  45
## 5          run  30
```

1. Get technical replicates.
2. Agglomerate at the Species rank.

```
tr <- dipp %>%
 subset_samples(TechReps) %>%
 tax_glom(taxrank = 'Species') %>%
 psmelt()
```

Calculate % standard deviation as a function of relative abundance.

```
mvar <- tr %>%
    ddply(~sample_id + Species + TechRep_type, function(x) {
      c(mean=mean(x$Abundance),
        var=var(x$Abundance),
        median=median(x$Abundance),
        sd=sd(x$Abundance),
        n=nrow(x),
        e=(qnorm(0.975)*sd(x$Abundance))/sqrt(nrow(x)))
    })
```

Compare % standard deviation across different types of technical replicates.

```
ggplot(mvar,
       aes(x=TechRep_type,
           y=sd/median,
           fill = TechRep_type)) +
geom_boxplot() +
scale_y_log10() +
xlab('source of variance') +
theme(legend.position='none')
```

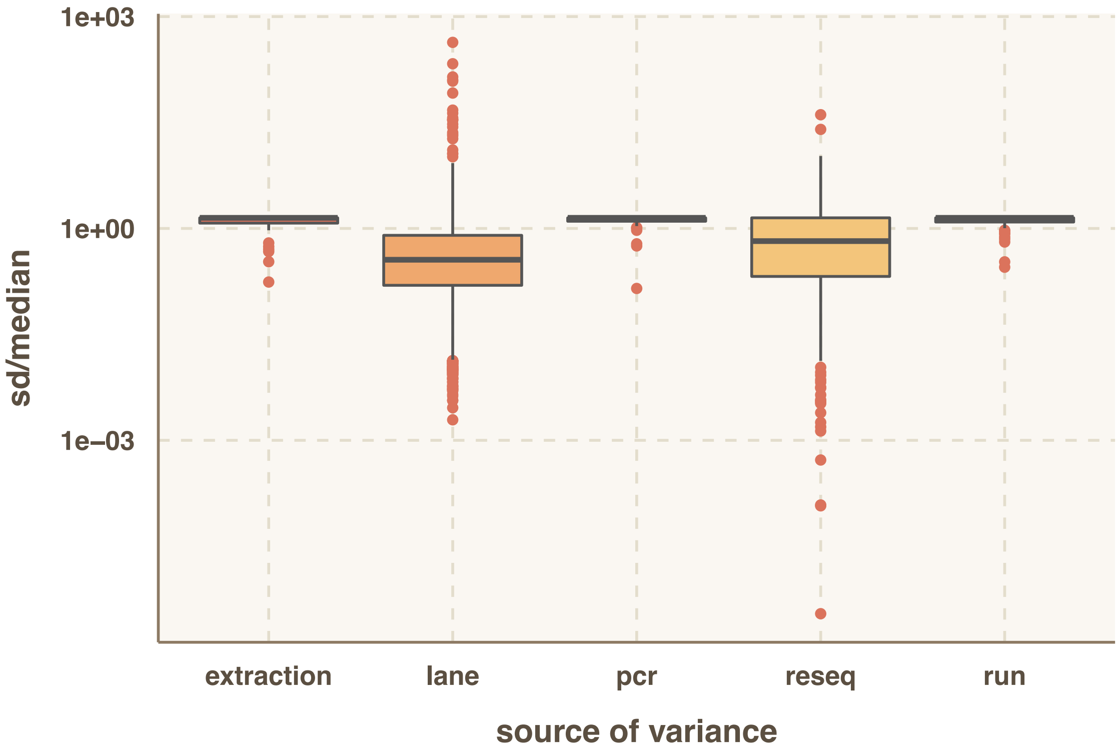

Plot variance as a function of relative abundance.

```
mvar <- mvar[complete.cases(mvar),]
mvar <- mvar[mvar$median > 0,]

ggplot(mvar,
       aes(x=median,
           y=sd/median)) +
geom_point() +
geom_vline(x=0.01) +
ggtitle('limit of quantification')
```

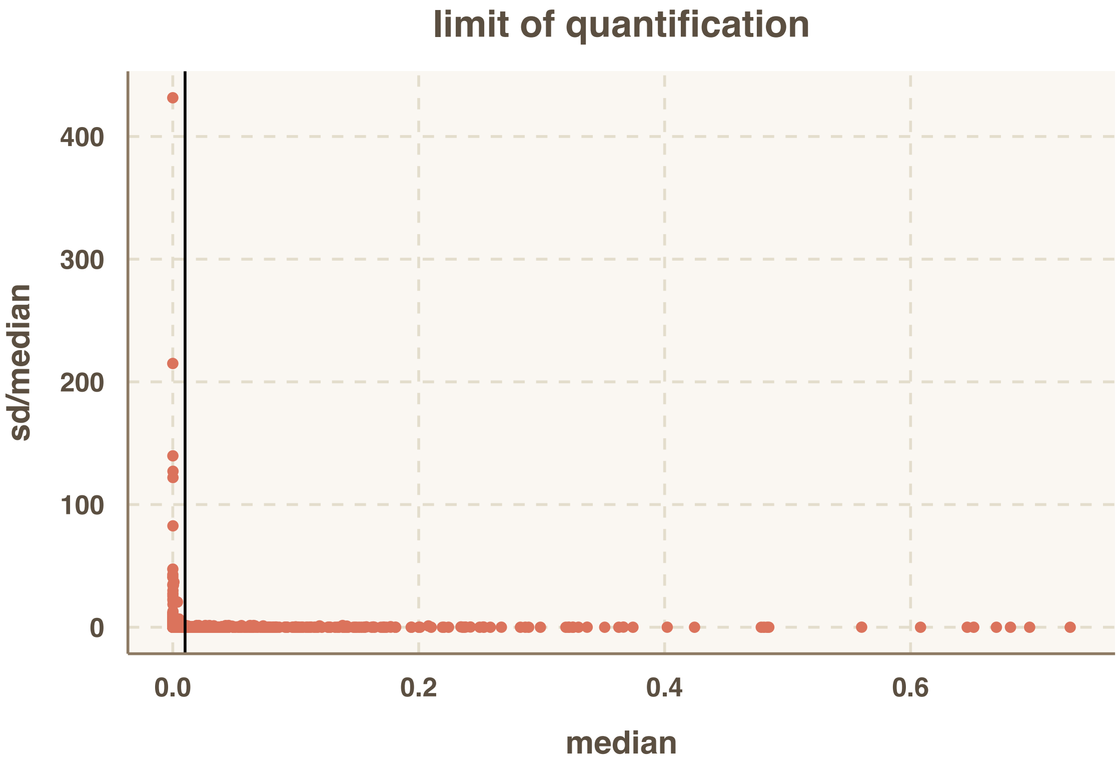

It looks like 1% isn't a bad cutoff. Below that, % standard deviation skyrockets.
