# Supplementary figures and images for: Bacteroides dorei dominates gut microbiome prior to autoimmunity in Finnish children at high risk for type 1 diabetes
Source: Front Microbiol. 2014 Dec 10;5:678. doi: 10.3389/fmicb.2014.00678 (PMC4261809; doi:10.3389/fmicb.2014.00678)

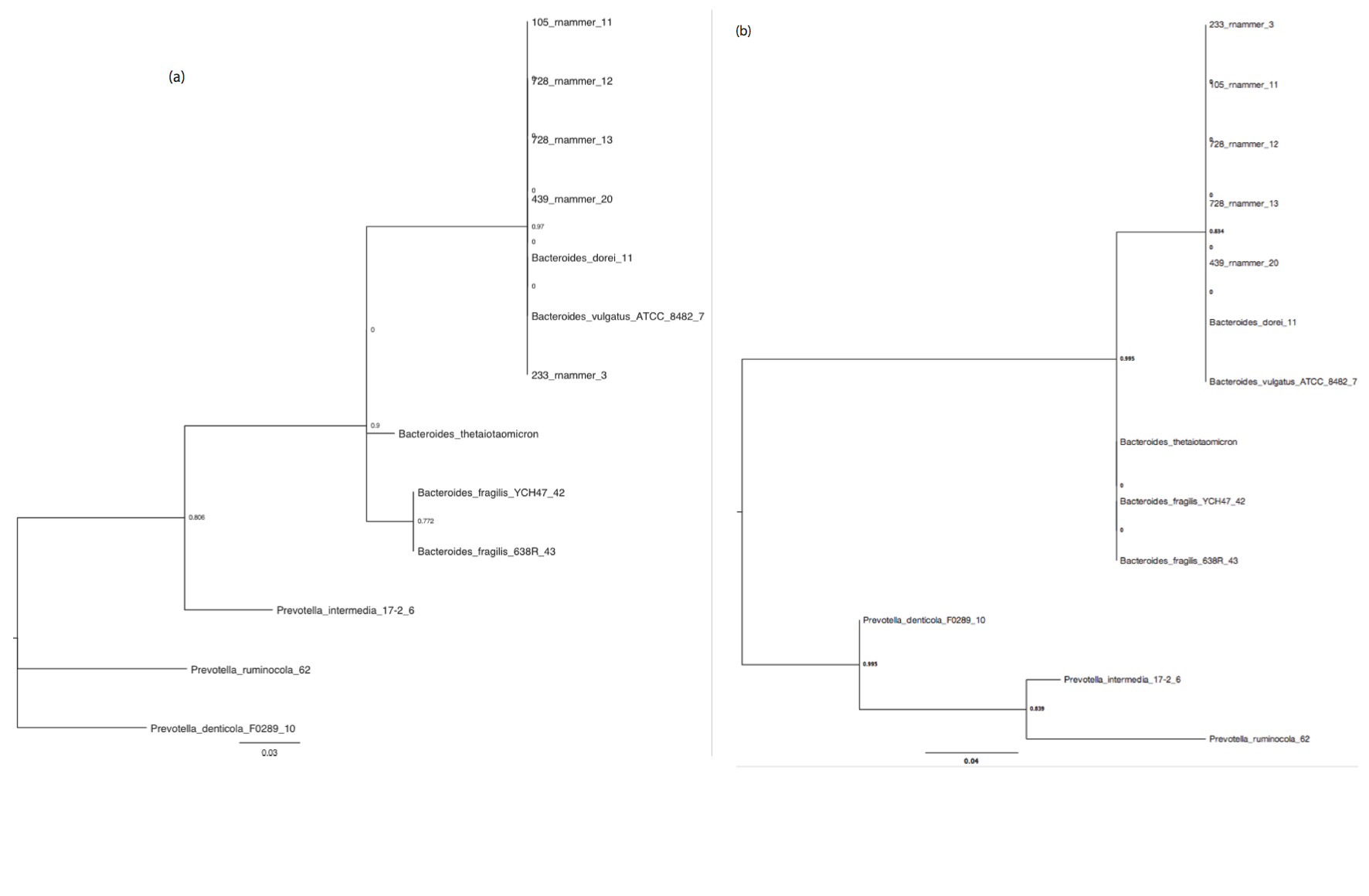

Supplement: Supplementary file 12 [file Image1.PNG]
